# *Arabidopsis* DNA repair mutants can integrate *Agrobacterium* T‐DNA into the plant genome

**DOI:** 10.1111/nph.71308

**Published:** 2026-06-01

**Authors:** Lan‐Ying Lee, Yunjia Shen, Yooyoung Kim, Ayako Nishizawa‐Yokoi, Hiroaki Saika, Demi White, Wenying Liao, Stanton B. Gelvin

**Affiliations:** ^1^ Department of Biological Sciences Purdue University West Lafayette IN 47907‐1392 USA; ^2^ Center for Plant Biology Purdue University West Lafayette IN 47907‐1392 USA; ^3^ Institute of Agrobiological Sciences National Agriculture and Food Research Organization 3‐1‐3 Kannondai Tsukuba 305‐8604 Japan; ^4^ Division of Plant Science and Technology University of Missouri Columbia MO 65211 USA

**Keywords:** *Agrobacterium*, *Arabidopsis*, nonhomologous end‐joining, plant transformation, T‐DNA integration

## Abstract

- *Agrobacterium* transfer DNA (T‐DNA) integration is mediated by plant DNA repair proteins of various nonhomologous end‐joining (NHEJ) pathways. However, the relative importance of these proteins for stable transformation of *Arabidopsis thaliana* is controversial.
- Using quantitative transient and stable transformation assays and droplet digital PCR analyses, we characterized single and higher order *Arabidopsis* NHEJ mutants to determine the importance of numerous NHEJ proteins in transformation and T‐DNA integration.
- Simultaneous mutation of genes important for several DNA repair/recombination pathways may only partially inhibit stable *Agrobacterium*‐mediated transformation. Some of these mutations may also inhibit transient transformation.
- These observations support the hypothesis that no individual NHEJ protein is essential for T‐DNA integration.

## Graphical abstract

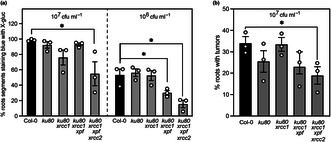

## Introduction

Virulent strains of *Agrobacterium* transfer DNA (T‐DNA) and Virulence (Vir) effector proteins to plants where T‐DNA, presumably in complexes with Vir and plant proteins, traverses the cytoplasm, enters the nucleus, and may integrate into the host genome (Gelvin, 2003, 2010, 2017, 2021; Pitzschke & Hirt, 2010; Lacroix & Citovsky, 2013, 2019; Kado, 2014; Nester, 2015; Shen & Lee, 2022; Thomson *et al*., 2023). Both before and following integration, transgenes carried on T‐DNA may be expressed.

The mechanism of T‐DNA integration remains an active topic of study. Several models have been proposed (Mayerhofer *et al*., 1991; Tinland, 1996; Tzfira *et al*., 2004; Saika *et al*., 2014; van Kregten *et al*., 2016). Because T‐DNA enters the plant cell as a single‐strand (ss) DNA molecule (T‐strand), which is guided to the nucleus by the covalently attached VirD2 protein, T‐DNA at some point in the integration process must be converted to a double‐strand (ds) form (Herrera‐Estrella *et al*., 1990; Tinland *et al*., 1994; Yusibov *et al*., 1994; Mysore *et al*., 1998; Ziemienowicz *et al*., 2001; Tao *et al*., 2004). When this occurs is not clear. One model posits that T‐strands ‘open up’ and ‘strand invade’ host DNA; a ss nick in the host genome allows ligation of T‐strands into the plant chromosome, following which second strand T‐DNA synthesis occurs (Mayerhofer *et al*., 1991). Although attractive, this model does not readily account for T‐DNA right border‐to‐right border (‘head‐to‐head’) or left border‐to‐left border (‘tail‐to‐tail’) dimer integration patterns that are frequently detected in transgenic organisms (Cluster *et al*., 1996). In addition, this model does not explain transient expression of T‐DNA encoded genes. Another model posits that T‐strands are first converted into double‐strand form in the nucleus before integration, and that these dsT‐DNA molecules are subsequently ligated into dsDNA breaks in the host genome (Salomon & Puchta, 1998; Chilton & Que, 2003; Tzfira *et al*., 2003, 2004; Liang & Tzfira, 2013; Dafny‐Yelin *et al*., 2015). Head‐to‐head or tail‐to‐tail ligation of these molecules before integration results in the corresponding integrated dimers. Additionally, ds circular T‐DNA molecules (‘T‐circles’) can form in *Agrobacterium*‐infected cells (Singer *et al*., 2012, 2022); it is not clear, however, whether T‐circles are a substrate for integration or a ‘dead‐end’ process that does not lead to T‐DNA integration. Recent evidence suggests that T‐DNA circles can integrate into the genome (Ye *et al*., 2024).

More recently, van Kregten *et al*. (2016) claimed that DNA polymerase θ is essential for T‐DNA integration. These authors presented a model by which the 3′ region of T‐strands hybridizes to resected ends near host DNA break sites. DNA polymerase θ subsequently replicates T‐strand sequences into the plant genome using the host DNA as a primer. Capture of two T‐strands on opposite sides of the break results in copying two T‐strand sequences into the genome; subsequent ligation of these sequences can form head‐to‐head T‐DNA dimers (van Kregten *et al*., 2016; Kralemann *et al*., 2022). However, other evidence indicates that DNA polymerase θ is not required for T‐DNA integration into somatic cell genomes of *Arabidopsis* and rice (Nishizawa‐Yokoi *et al*., 2021; Nishizawa‐Yokoi & Gelvin, 2023).

The potential significance of dsDNA breaks in T‐DNA integration has led to several studies on the importance of plant dsDNA break repair/recombination enzymes in stable *Agrobacterium*‐mediated transformation. Studies investigating the role of *Arabidopsis* and rice genes encoding Ku70, Ku80, or DNA ligase IV (*lig4*) have come to varying conclusions. Some have indicated that mutation of these genes lowers the frequency of stable transformation (Friesner & Britt, 2003; Li *et al*., 2005; Jia *et al*., 2012; Nishizawa‐Yokoi *et al*., 2012; Mestiri *et al*., 2014; Saika *et al*., 2014), whereas others have concluded that these mutations have no or little effect on stable transformation (Gallego *et al*., 2003; van Attikum *et al*., 2003). Kralemann *et al*. (2022) presented evidence that Ku80 may be involved in integrating T‐DNA right borders into the plant genome. Additionally, Furukawa *et al*. (2015) presented evidence of a role for DNA polymerase lambda (Polλ) in *Arabidopsis* stable transformation.

In *Arabidopsis*, most investigations used a flower‐dip protocol to measure transformation (Friesner & Britt, 2003; Gallego *et al*., 2003; van Attikum *et al*., 2003; Jia *et al*., 2012; Mestiri *et al*., 2014; Furukawa *et al*., 2015). However, because flower‐dip transformation can readily occur in *Arabidopsis rat* (resistant to *Agrobacterium*‐mediated transformation) mutant plants that display greatly reduced root transformation, these flower‐dip transformation studies may not reflect the T‐DNA integration process that occurs in somatic cells (Nam *et al*., 1999; Mysore *et al*., 2000a; Zhu *et al*., 2003). In addition, because T‐DNA integration is affected by numerous genes that may also influence transgene expression (Crane & Gelvin, 2007), integration can occur in the absence of transgene expression (Park *et al*., 2015). Thus, lack of stable transformation, an assay based on transgene expression, may not indicate lack of T‐DNA integration.

Silencing of *Nicotiana benthamiana* or *Arabidopsis XRCC4*, a gene encoding a protein important for canonical nonhomologous end‐joining (C‐NHEJ), resulted in both increased stable transformation and T‐DNA integration into the host genome (Vaghchhipawala *et al*., 2012). Similarly, mutation of *Arabidopsis Ku70*, *Ku80*, or the gene encoding DNA ligase VI (*lig6*) increased both of these processes using a root transformation assay (Park *et al*., 2015). Mutation of *PARP1*, a gene involved in alternative NHEJ (A‐NHEJ), resulted in increased T‐DNA integration into the host genome in the absence of increased stable transformation due to high levels of T‐DNA methylation and, presumably, transgene silencing (Park *et al*., 2015). These studies suggest that mutation of NHEJ genes may slow the DNA break repair process, thus providing more opportunity for integration of T‐DNA into genomic dsDNA breaks.

Mestiri *et al*. (2014) investigated single and multiple (higher order) mutations in *Arabidopsis* genes, including *ku80* (C‐NHEJ), *xrcc1* (A‐NHEJ), *xpf* (involved in many forms of repair, including A‐NHEJ and homologous recombination (HR)), and *xrcc2* (HR), important for known DNA repair/recombination pathways. With increasing numbers of mutations, stable *Agrobacterium*‐mediated transformation substantially decreased, although it was not eliminated in the quadruple mutant. The authors concluded that these genes (and pathways) are important for stable transformation; however, some additional pathway must exist to explain the residual level of stable transformation of the quadruple mutant.

We further characterized these and other mutants, all null mutants with T‐DNA insertions in the coding regions, to determine the efficiency of T‐DNA integration into the host genome. Considering the reported roles for DNA polymerase θ and for DNA polymerase λ in T‐DNA integration (Furukawa *et al*., 2015; van Kregten *et al*., 2016), we generated mutant *Arabidopsis* lines incorporating these mutations with other NHEJ gene mutations. Our results indicate that as increasing numbers of DNA repair/recombination genes are mutated, plant growth can decline substantially, especially when plants are grown under high light conditions. In parallel, transient transformation can, in some instances, decrease. However, stable transformation can still occur. These results support our hypothesis that no one known DNA repair/recombination pathway is essential for T‐DNA integration, although individually and collectively they may affect the extent of integration and stable transformation.

## Materials and Methods

### Bacterial and plant growth

*Escherichia coli* were grown at 37°C in Luria‐Bertani (LB) medium (Sambrook *et al*., 1989). *Agrobacterium tumefaciens* were grown at 28°C in either YEP rich medium or AB minimal medium (Lichenstein & Draper, 1986). When appropriate, the following antibiotics were used (μg ml^−1^): for *E. coli*, ampicillin, 50; kanamycin, 25; for *A. tumefaciens*, rifampicin, 10; kanamycin, 100 (solidified medium) or 50 (liquid medium; Zhu *et al*., 2003). *Arabidopsis thaliana* (L.) seeds were surface sterilized with 50% bleach, 0.1% sodium dodecylsulfate (SDS) and germinated at 25°C in Petri dishes on solidified Gamborg's B5 medium (Caisson Laboratories, Logan, UT, USA, http://www.caissonlabs.com) containing 100 μg ml^−1^ Timentin and grown under normal (*c*. 150 μEinsteins m^−2^ s^−1^) or low (*c*. 9 μEinsteins m^−2^ s^−1^) light, long‐day (14 h light) conditions at 25°C. Plants were moved to baby food jars containing solidified B5 medium and grown for a further 3 wk under normal or low light conditions. For growth experiments, surface sterilized seeds were plated on Petri dishes containing B5 medium plus Timentin and grown vertically under normal or low light conditions.

### Plant materials

The following mutants were used in this study: *ku80* (SAIL_714_A04); *xrccI* (SALK_125373C); *polλ* (SALK_075391C); and *polQ* (*teb2*, SALK_035610C). Crosses were made among these mutant lines and homozygous F2 progeny confirmed by PCR. Some higher order mutants were obtained from Dr. Charles White. Because the *ku80* mutant and derived higher order mutant plants contain a nonfunctional *gusA* gene, we did not conduct dd‐PCR of the *gusA* gene to determine nonselective T‐DNA integration with these mutants.

### Transient and stable transformation assays

Transformation assays were conducted as described previously (Nam *et al*., 1999; Mysore *et al*., 2000a; Zhu *et al*., 2003). Briefly, transient transformation assays were performed using *A. tumefaciens* At849, a nononcogenic strain containing the T‐DNA binary vector pBISN1 (Narasimhulu *et al*., 1996), or *A. tumefaciens* At802 (the tumorigenic strain *A. tumefaciens* A208 containing pBISN1). pBISN1 contains a *gusA*‐intron gene that expresses in plants but not in bacteria. *Arabidopsis* root segments were assayed 6 d after infection. Stable transformation was measured using the tumorigenic strain *A. tumefaciens* A208 or *A. tumefaciens* At802, assaying for the formation of crown gall tumors 1 month after infection.

### T‐DNA integration assay

Incorporation of T‐DNA was measured as described previously (Mysore *et al*., 1998; Crane & Gelvin, 2007; Park *et al*., 2015). Briefly, *Arabidopsis* root segments were inoculated with *A. tumefaciens* At849. Two days later, the segments were transferred to solidified callus inducing medium (CIM) medium (Nam *et al*., 1997) containing 100 μg ml^−1^ Timentin, and calli were grown for 1 month. The calli were transferred to fresh CIM medium containing 100 µg ml^−1^ Timentin and grown for a further one month before DNA extraction.

### Droplet digital PCR

Genomic DNA, extracted from *Arabidopsis* calli using a Viogene Plant Genomic DNA Miniprep System, was digested with *Sna*BI. T‐DNA copy number evaluation and detection of *Agrobacterium* contamination by dd‐PCR was performed as in Nishizawa‐Yokoi *et al*. (2021). We used PrimePCR™ Probe Assay: *ACT2*, *Arabidopsis* labelled with HEX (Bio‐Rad) for normalization (Vaghchhipawala *et al*., 2018; Giraldo *et al*., 2019; Ziegler *et al*., 2019). As a control for *Agrobacterium* DNA contamination in the samples, we also conducted dd‐PCR using primers directed against the *Agrobacterium lipA* chromosomal gene. Gene‐specific primers were:

*hptII*‐F (ATTTCGGCTCCAACAATGTC), *hptII*‐R (AGATGTTGGCGACCTCGTAT), *hptII*‐FAM probe (TTGACTGGAGCGAGGCGATGTTC); *lipA*‐F (AGCCTTCCACGCCTTTCCT), *lipA*‐R (CCGCTTTTCCCGACGAT), *lipA*‐FAM probe (CCACCTTGCAGATTG).

### Statistical analysis

Where applied, Steel's test was used to compare each mutant sample with the control (Columbia‐0 (Col‐0)) with the statistical software EZR (Easy R; Kanda, 2013); significant differences are indicated with asterisks (*P* < 0.05).

## Results

### Germination and growth of multiple DNA repair/recombination mutants in normal and low light growth conditions

We obtained seeds of homozygous single (*ku80*), double (*ku80*/*xrcc1*), triple (*ku80*/*xrcc1*/*xpf*), and quadruple (*ku80*/*xrcc1*/*xpf*/*xrcc2*) DNA repair/recombination mutants from the Charles White laboratory (Mestiri *et al*., 2014) and germinated them, along with wild‐type (WT) Col‐0 seeds from the Gelvin and White seed stock collections, on B5 medium under ‘normal’ (*c*. 150 μEinsteins m^−2^ s^−1^) or low (*c*. 9 μEinsteins m^−2^ s^−1^) light conditions (recommended growth condition is 120–150 μEinsteins m^−2^ s^−1^; https://abrc.osu.edu/seed‐handling). Under normal light conditions, all WT seeds germinated, but with an increasing number of DNA repair/recombination mutations, a lower percentage of seeds germinated (Supporting Information Fig. S1A; relevant data for experiments are presented in Dataset S1). We obtained similar germination results for seeds germinated under low light conditions (Fig. S1B).

Shoot and root growth of DNA repair/recombination mutants under normal light conditions was poor relative to that of WT plants, and increasing the number of these mutations resulted in progressively poorer growth (Fig. S1A). Growth of the triple and quadruple mutants was especially slow; of the relatively few seeds of these mutants that germinated, flowering initiated when the plants had few leaves. However, when these mutants were germinated and grown under low light conditions, both shoot and root growth appeared normal compared with WT plants (Fig. S1B). Plants harboring mutations in multiple DNA repair pathways may have difficulty repairing mutations induced by normal light conditions and may therefore accumulate mutations detrimental to seed germination, growth, and development (Britt, 1996; Waterworth *et al*., 2010; Manova & Gruszka, 2015). Indeed, crossing a poorly growing homozygous *ku80* mutant plant to a homozygous *parp1* mutant plant resulted in normal growth and development of the resulting homozygous *ku80*/*parp1* F2 progeny (Park *et al*., 2015), suggesting that homozygous mutations deleterious to plant growth and development had been eliminated.

### Transformation of DNA repair/recombination mutants grown under various light conditions

**Fig. 1:**
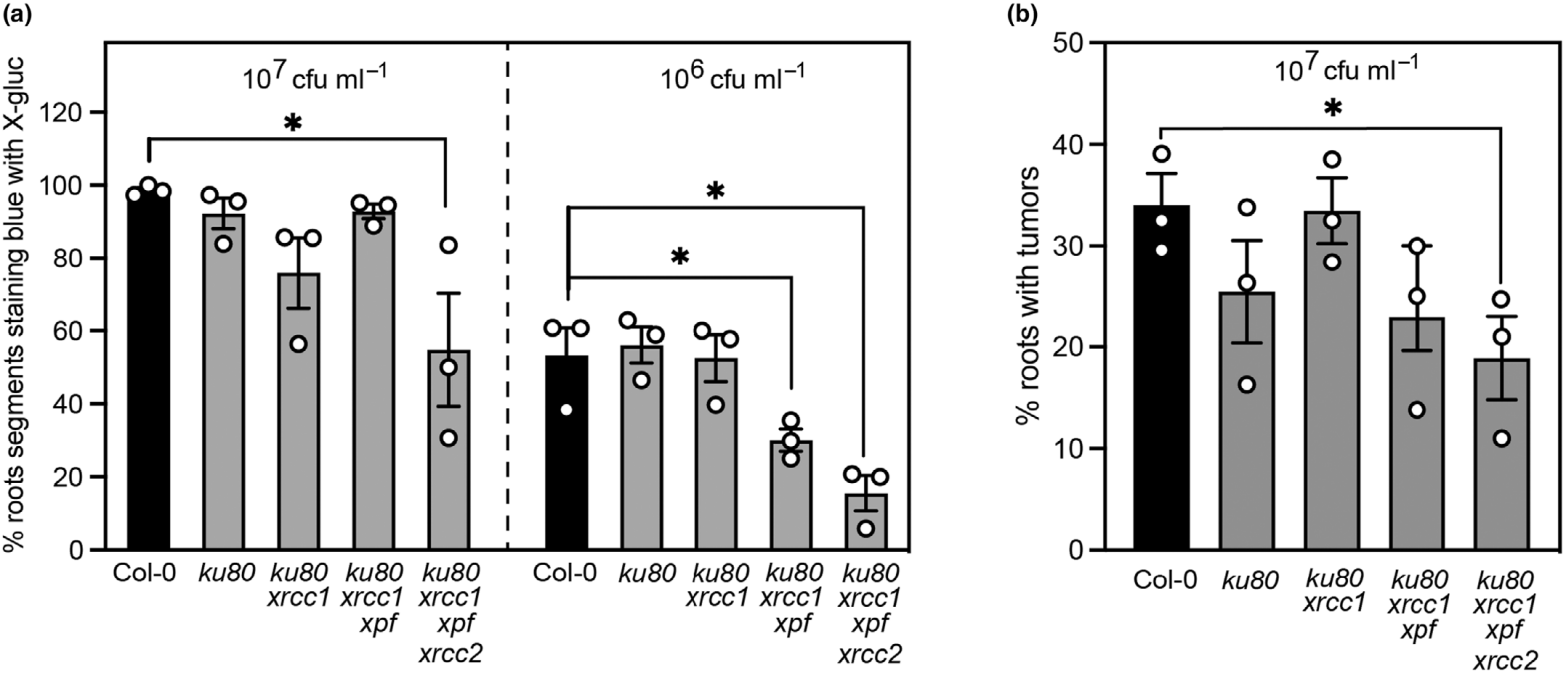
Transient (a) and stable (b) transformation of *Arabidopsis thaliana* DNA repair/recombination mutants grown under normal light conditions. (a) Segments from pooled roots of 5–10 plants were inoculated with *A. tumefaciens* At849, containing the transfer DNA (T‐DNA) binary vector pBISN1, at the indicated concentration, then cocultivated on Murashige and Skoog (MS) medium. After 2 days, the segments were moved to plates containing callus inducing medium (CIM) plus 100 μg ml^−1^ Timentin and incubated for an additional 4 d. Following X‐gluc staining, the root segments were visualized using a dissecting microscope and scored for the presence of GUS activity. For each assay point, 130–600 root segments were scored. Wild‐type (WT) Columbia‐0 (Col‐0) seeds were from the White laboratory. (b) Segments from pooled roots of 5–10 plants were inoculated with the tumorigenic strain *A. tumefaciens* A208 at a concentration of 10^7^ CFU ml^−1^, then cocultivated on MS medium. After 2 days, the segments were moved to plates containing MS medium plus 100 μg ml^−1^ Timentin. Root segments were scored for tumor formation after 1 month. For each assay point, 240 root segments were scored. WT Col‐0 seeds were from the White laboratory. Data are mean ± SE (*n* = 3); significant differences (*, *P* < 0.05) based on Student's *t*‐test compared with Col‐0 are indicated by asterisks.

We first assayed WT and mutant plants grown under normal light conditions for transient and stable transformation, using well‐established quantitative assays (Nam *et al*., 1997; Zhu *et al*., 2003; Tenea *et al*., 2009). Transient transformation was measured as the percentage of root segments staining blue with 5‐bromo‐4‐chloro‐3‐indolyl‐β‐d‐glucuronic acid (X‐gluc) 6 d after inoculation with *A. tumefaciens* At849, which harbors a *gusA*‐intron gene within the T‐DNA of the binary vector pBISN1 (Narasimhulu *et al*., 1996). At high bacterial inoculation conditions (10^7^ CFU ml^−1^), root segments of the triple mutant showed equivalent transformation efficiency to that of WT plants, whereas segments of the quadruple mutant showed 56% the transformation efficiency as that of WT plants (Fig. 1a), a 1.8‐fold reduction in transformation efficiency. This latter number is similar (61%; 1.6‐fold reduction) to that obtained by Mestiri *et al*. (2014) for the quadruple mutant, using a very high bacterial inoculum (2 × 10^8^ CFU ml^−1^). However, when we inoculated root segments with a lower bacterial inoculum (10^6^ CFU ml^−1^), the triple and quadruple mutants showed considerably lower transient transformation efficiency than did WT plants (56% and 29%, respectively, equivalent to 1.8‐ and 3.5‐fold reductions). The greater reduction in transformation efficiency at lower bacterial inoculum reflects the lack of ‘saturation’ shown when high bacterial inocula are used. Further reduction in the bacterial inoculum to 10^5^ CFU ml^−1^ resulted in transformation efficiency numbers too low to be reliable. Because the presumed effect of the DNA repair/recombination mutants would be on T‐DNA integration, a property not measured by transient transformation assays (Mysore *et al*., 1998), the large decrease in transient transformation frequency shown by the triple and quadruple mutants may reflect the diminished ‘health’ of these mutants when grown under normal light conditions (Fig. S1A).

We assayed susceptibility to stable *Agrobacterium*‐mediated transformation by inoculating root segments of WT and DNA repair/recombination mutants with the tumorigenic strain *A. tumefaciens* A208, using a standard crown gall tumorigenesis assay (Zhu *et al*., 2003). Interestingly, the triple and quadruple mutants showed relatively high stable transformation frequencies (67% and 56% that of WT, respectively; Fig. 1b).

Quantitative root transformation assays conducted in our laboratory routinely use plants grown under normal light conditions (e.g. Zhu *et al*., 2003; Mysore *et al*., 2000a, 2000b; Hwang & Gelvin, 2004; Crane & Gelvin, 2007; Bhattacharjee *et al*., 2008; Tenea *et al*., 2009; Sardesai *et al*., 2013). However, because of concern that normal light levels may impair the growth of DNA repair/recombination mutants and subsequently alter their susceptibility to *Agrobacterium*‐mediated transformation, we grew and assayed selected mutants under low light conditions that did not impair growth (Fig. S1B).

Despite normal growth of roots under low light conditions, transient transformation efficiencies of the triple and quadruple mutants were greatly decreased. Relative to that of WT root segments, these mutants showed 18.0% and 9.7% transient transformation efficiency, equivalent to 5.6‐ and 10.3‐fold decreases (Fig. S2A). These results indicate either that these DNA repair/recombination genes are important for transient transformation, or that other mutations accumulating in these mutant lines inhibited cellular transformation processes other than T‐DNA integration.

Similar to the situation with plants grown under normal light conditions, mutant plants grown and assayed under low light conditions showed a relatively small decrease in stable transformation. When assayed at a bacterial inoculum of 10^7^ CFU ml^−1^, the triple and quadruple mutants showed, relative to WT, 53% and 49.1% stable transformation efficiency, respectively, equivalent to approximately twofold decreases (Fig. S2B).

We conclude that none of the genes mutated in these lines are essential for transformation, and both individually and collectively may only be quantitatively important for T‐DNA integration.

### T‐DNA integration into the genomes of wild‐type and DNA repair/recombination mutants

The extent of T‐DNA integration into the plant genome may reflect the stable transformation efficiency. However, T‐DNA integration can occur without transgene expression; thus, integration and stable transformation assay results may not correlate with each other. For example, an *Arabidopsis parp1* mutant showed transient and stable transformation frequencies similar to those of WT plants, but T‐DNA integration into the genome of the *parp1* mutant was more extensive than integration into the genome of WT plants. This lack of correlation between integration and stable transformation assay results may be attributed to the higher degree of DNA methylation, and subsequent silencing, of integrated transgenes in the *parp1* genome (Park *et al*., 2015). We therefore directly assayed the extent of T‐DNA integration into WT and DNA repair/recombination mutant genomes in the absence of selection for the expression of transgenes. Similar protocols were used to assess the extent of T‐DNA integration into the genomes of WT and mutant *Arabidopsis* plants (Mysore *et al*., 2000b; Anand *et al*., 2007; Crane & Gelvin, 2007; Vaghchhipawala *et al*., 2012; Park *et al*., 2015; Nishizawa‐Yokoi *et al*., 2021).

We inoculated root segments of WT and several DNA repair/recombination mutants, grown under low light conditions, with *A. tumefaciens* At2093 (the disarmed, nontumorigenic strain *A. tumefaciens* GV3101::pMP90 containing a T‐DNA binary vector encoding *hptII* and *gusA*‐intron genes) at two bacterial concentrations, 10^8^ and 10^9^ CFU ml^−1^. After 2 days cocultivation, we grew calli from these root segments on solidified CIM containing Timentin (to kill *Agrobacterium*) but without selection for transgene expression for 2 months, transferring the calli to fresh Timentin‐containing CIM after the first month of growth. We subsequently pooled a minimum of 50 calli for DNA extraction and conducted droplet digital (dd)‐PCR to quantify the amount of T‐DNA incorporated into the genome of each genotype. As a control for bacterial contamination of the plant samples, we also conducted dd‐PCR using primers directed against the *Agrobacterium lipA* chromosomal gene.

**Fig. 2:**
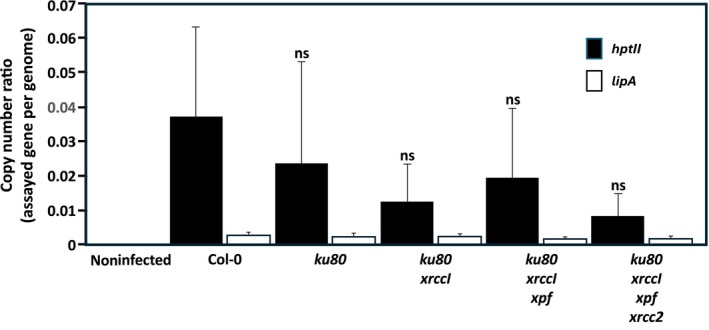
Integrated transfer DNA (T‐DNA) copy number in wild‐type and nonhomologous end‐joining (NHEJ) mutant *Arabidopsis thaliana* calli after growth under nonselected conditions. Root segments from the indicated *Arabidopsis* genotypes were inoculated with *A. tumefaciens* At2093 containing *gusA‐*intron and *hptII* genes in the T‐DNA, at the indicated bacterial concentrations. After 2 days cocultivation the root segments were transferred to callus inducing medium (CIM) containing timentin but no hygromycin. After 1 month, the emerging calli were again transferred to CIM plus timentin and grown for one more month. For each set of samples, DNA was extracted from a minimum of 50 pooled calli and subjected to dd‐PCR analysis using primers directed against the *hptII* gene. Error bars indicate SE. ns, not signficant.

Fig. 2 shows that, in general, the amount of T‐DNA incorporated into plant DNA was approximately the same, or slightly less, in all the mutants as in WT plant DNA (*c*. 1 copy per 100–200 plant genomes). These experiments indicate that T‐DNA can integrate into the genomes of plants simultaneously lacking up to four different DNA repair and recombination pathways. The data further show that as these pathways are disrupted, T‐DNA integration may slightly decrease relative to that of WT plants. These results are consistent with the hypothesis that T‐DNA integration does not absolutely require these DNA repair pathways.

### Transformation and T‐DNA integration into the genomes of *Arabidopsis* plants harboring DNA polymerase θ mutations

DNA polymerase θ is important for T‐DNA integration, although we have shown that it is not essential for transformation and integration into the genomes of *Arabidopsis* and rice somatic cells (van Kregten *et al*., 2016; Nishizawa‐Yokoi *et al*., 2021; Kralemann *et al*., 2022; Nishizawa‐Yokoi & Gelvin, 2023). We were curious as to whether combining a *polQ* mutation with mutations in other DNA repair genes would affect *Agrobacterium*‐mediated transformation and T‐DNA integration. We therefore incorporated the *polQ* mutation *teb2* into the genomes of *ku80* and *ku80/xrccI* mutant *Arabidopsis* plants and used these plants for transient and stable transformation assays.

**Fig. 3:**
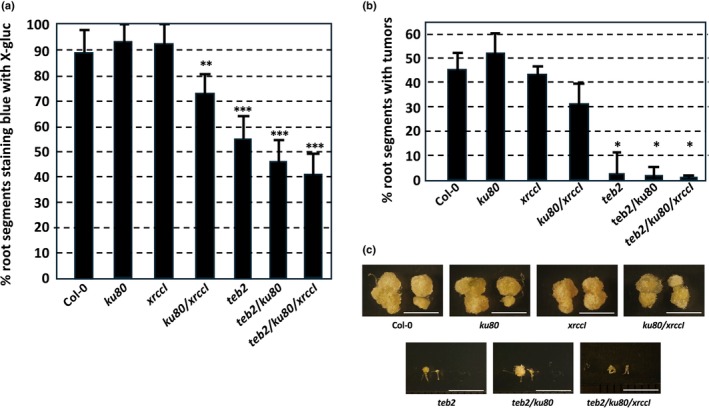
Transient and stable transformation of *Arabidopsis thaliana* nonhomologous end‐joining (NHEJ) single and higher order mutants. (a) Root segments (> 150) were infected and cocultivated with *A. tumefaciens* At849 containing a *gusA*‐intron gene within the transfer DNA (T‐DNA) at 10^8^ CFU ml^−1^. After 2 d the roots were transferred to callus inducing medium (CIM) medium containing Timentin and incubated for an additional 4 d, following which they were stained with X‐gluc. Data are mean ± SE (*n* = 3); significant differences (**, *P* < 0.01; ***, *P* < 0.001) based on Student's *t*‐test compared with Columbia‐0 (Col‐0) are indicated by asterisks. (b) Root segments (> 125) were inoculated with the tumorigenic strain *A. tumefaciens* A208 at 10^8^ CFU ml^−1^. After 2 d the root segments were transferred to Murashige and Skoog (MS) medium containing Timentin and incubated for an additional 1 month. Data are mean ± SE (*n* = 3); significant differences (*, *P* < 0.05) based on Student's *t*‐test compared with Col‐0 are indicated by asterisks. (c) Photographs of tumors after 1 month growth. Bars, 5 mm.

Transient transformation assays using *A. tumefaciens* At849 (*A. tumefaciens* GV3101::pMP90 containing the T‐DNA binary vector pBISN1) indicated that GUS (β‐glucuronidase) activity was similar among the WT, *ku80*, and *xrccI* mutants. However, transient GUS activity was diminished in the *ku80*/*xrccI*, *polQ*, *polQ*/*ku80*, and *polQ*/*ku80*/*xrcc1* mutants (Fig. 3a). The decrease in transient transformation of the *polQ* mutant suggests that DNA polymerase θ may be involved in steps of the transformation process other than just T‐DNA integration.

Stable transformation assays, using the tumorigenic strain *A. tumefaciens* A208, indicated a slight transformation increase in the *ku80* mutant relative to WT plants at high bacterial concentration (10^8^ CFU ml^−1^), as seen previously (Fig. 3b; Park *et al*., 2015). All tested genotypes containing the *polQ* mutation had low but detectable stable transformation frequencies, as described previously (Nishizawa‐Yokoi *et al*., 2021). In addition, the tumors were smaller and grew more slowly (Fig. 3c).

**Fig. 4:**
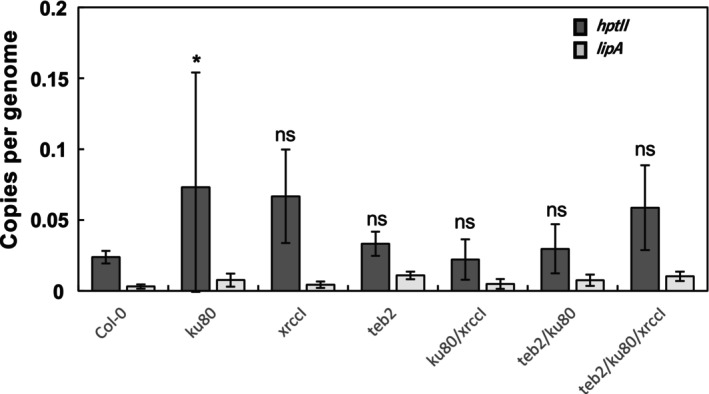
Integrated transfer DNA (T‐DNA) copy number in wild‐type (WT), NHEJ, and *polQ* mutant *Arabidopsis thaliana* calli after growth under nonselected conditions. Root segments from the indicated *Arabidopsis* genotypes were infected with *A. tumefaciens* At2308 containing a *hptII* gene in the T‐DNA at 10^9^ CFU ml^−1^. After 2 d cocultivation the root segments were transferred to callus inducing medium (CIM) containing Timentin but no hygromycin. After 1 month, the emerging calli were again transferred to CIM plus Timentin and grown for one more month. For each set of samples DNA was extracted from a minimum of 50 pooled calli and subjected to dd‐PCR analysis using primers directed against the *hptII* or chromosomal *lipA* genes. Data are mean ± SE (*n* ≥ 3); significant differences (*, *P* < 0.05) are indicated by asterisks, based on Steel's test, when each mutant sample is compared with Columbia‐0 (Col‐0). Samples with a *lipA* value of more than 0.015/genome were excluded from the analysis, as these were considered contaminated by *Agrobacterium*.

We further conducted dd‐PCR analysis to quantify T‐DNA integration into calli of these mutant plants grown under nonselective conditions. Fig. 4 shows that, corresponding to the increase in stable transformation and results previously published (Park *et al*., 2015), the *ku80* mutant showed a significant increase in T‐DNA integration relative to that seen with WT plants. However, none of the other mutants showed a significant difference in T‐DNA integration.

**Fig. 5:**
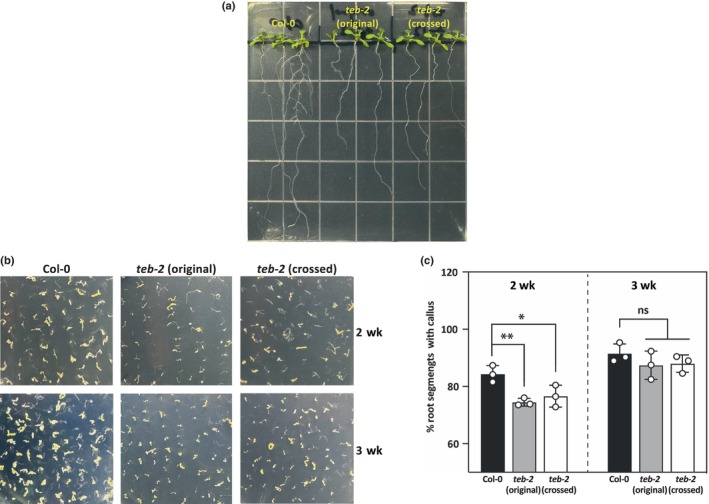
Growth of roots and root‐derived calli from wild‐type (WT) and *teb2* mutant *Arabidopsis thaliana* plants. (a) Seeds of WT Columbia‐0 (Col‐0) and homozygous *teb2* mutant plants before (original) and after (crossed) crossing to Col‐0 were plated onto solidified B5 medium containing 100 μg ml^−1^ Timentin and grown vertically for 10 d. (b) Root segments (3–5 mm) were cut from the indicated plants and plated onto solidified callus inducing medium (CIM) containing 100 μg ml^−1^ Timentin and grown for the indicated number of weeks. Note that *teb2* calli grow more slowly than do calli from WT plants. (c) Quantification of the percentage of root segments showing callus growth after the indicated number of weeks. Data are the mean + SE (*n* = 3); significant differences (*, *P* < 0.1; **, *P* < 0.01; ns, not significant) based on Student's *t*‐test compared with Col‐0 are indicated by asterisks.

**Fig. 6:**
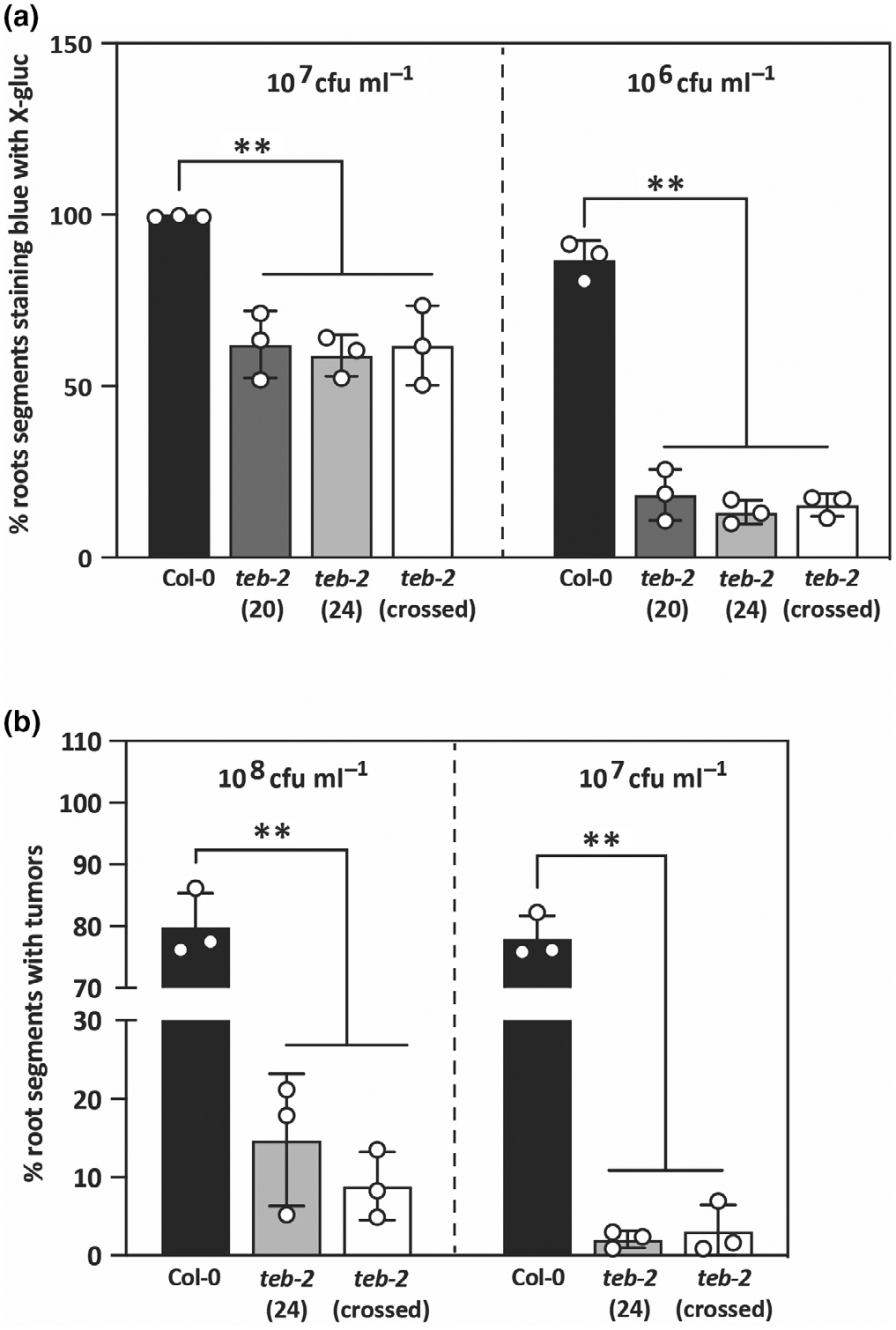
Transformation of *Arabidopsis thaliana* wild‐type (WT) and *teb2* mutant root segments. (a) Transient transformation. Root segments (> 250) were infected with *A. tumefaciens* At802 containing a *gusA*‐intron gene within the transfer DNA (T‐DNA) at the indicated bacterial concentrations. After 2 days cocultivation the roots were transferred to callus inducing medium (CIM) medium containing Timentin and incubated for an additional 4 d, following which they were stained with X‐gluc. (b) Stable transformation. Root segments (> 120) were inoculated with the tumorigenic strain *A. tumefaciens* At802 at the indicated bacterial concentrations. After 4 days, the roots were transferred to Murashige and Skoog (MS) medium containing Timentin and incubated for an additional 1 month. *teb2* (20), seeds collected from *teb2* plants in 2020; *teb2* (24), seeds collected from *teb2* plants in 2024; *teb2* (crossed), seeds of homozygous *teb2* plants after crossing with Columbia‐0 (Col‐0). Data are the mean ± SE (*n* = 3); significant differences (**, *P* < 0.01) based on Student's *t*‐test compared with Col‐0 are indicated by asterisks.

We had previously determined that calli grown from nontransformed *polQ* mutant roots grew more slowly than did calli from WT plants (Nishizawa‐Yokoi *et al*., 2021). Nisa *et al*. (2021) also noted pleiotropic effects of *Arabidopsis polQ* mutations on plant growth. We were concerned that the accumulation of secondary mutations, resulting from slower DNA repair, could contribute to the growth and transformation phenotypes of our *polQ* mutant lines. We therefore crossed our *teb2* mutant line with WT Col‐0 plants, then selfed the resulting heterozygous *polQ* mutant to obtain new homozygous *polQ* mutant lines, thus ‘diluting’ homozygous secondary mutations. Growth of the new mutant line closely resembled growth of the original *teb2* mutant in culture: compared with WT plants, the roots grew more slowly and were less branched (Fig. 5a). Calli from root segments of both *polQ* mutants grew more slowly than did calli from Col‐0 plants (Fig. 5b,c). Transient (Fig. 6a) and stable (Fig. 6b) transformation assays indicated that the transformation frequency of both the new and old *teb2* mutants was equivalent, and less than that of WT plants. Thus, the lower transient and stable transformation frequencies of *polQ* mutant plants are likely caused by the *PolQ* mutation and not by secondary background mutations.

### DNA polymerase λ (Polλ) mutants show increased transient and stable root transformation frequencies

Furukawa *et al*. (2015) showed that DNA polymerase λ may be involved in *Arabidopsis* flower‐dip transformation, and more recently Chandramouly *et al*. (2023) showed that human Polλ could participate in microhomology‐mediated end‐joining (MMEJ). Because of the known role for MMEJ, and the importance of PolQ in T‐DNA integration (van Kregten *et al*., 2016; Kralemann *et al*., 2022), we assayed transient and stable *Agrobacterium*‐mediated transformation of roots of *polλ* and *polλ*/*polQ* mutant *Arabidopsis* plants. For these studies, we used the same *polλ‐1* mutant used by Furukawa *et al*. (2015).

**Fig. 7:**
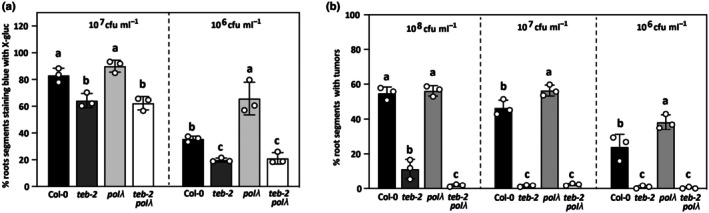
Transformation of *Arabidopsis thaliana* wild‐type (WT), *teb2*, and *polλ* mutant root segments. (a) Transient transformation. Root segments (> 220) were infected with *A. tumefaciens* At802 containing a *gusA*‐intron gene within the transfer DNA (T‐DNA) at the indicated bacterial concentrations. After 2 days, the roots were transferred to callus inducing medium (CIM) medium containing Timentin and incubated for an additional 4 d, following which they were stained with X‐gluc. (b) Stable transformation. Root segments (> 120) were infected with the tumorigenic strain *A. tumefaciens* At802 at the indicated bacterial concentrations. After 2 days, the roots were transferred to Murashige and Skoog (MS) medium containing Timentin and incubated for an additional 1 month. *teb2*/*polλ*, homozygous seeds of *teb2* plants after crossing with *polλ* plants. Data are the mean ± SE (*n* = 3) of independent biological replicates. Letters denote groups with significant differences from one‐way analysis of variance (ANOVA) followed by Tukey's Honestly Significant Difference test at *P* < 0.05.

Fig. 7a and b shows that, as opposed to the decrease in both transient and stable transformation frequencies of *polλ* mutant plants seen by Furukawa *et al*. (2015), *polλ* mutant plants were hyper‐susceptible to both transient and stable transformation. The *polλ*/*polQ* double mutant showed little difference in transformation frequency from that of the *polQ* single mutant. These data indicate that Polλ is involved in both T‐DNA integration and processes preceding integration during *Arabidopsis* root transformation.

## Discussion

T‐DNA integration and transgene expression are the last steps in stable *Agrobacterium*‐mediated transformation, although transient transformation (including transgene expression) may occur in the absence of T‐DNA integration. Understanding integration is important for scientists to control various aspects of integration: targeting T‐DNA integration to specific regions of the plant genome, reducing integrated T‐DNA copy number, a desired characteristic for commercial transgenic crops, and permitting efficient transient transformation in the absence of integration to deliver CRISPR reagents for transgene‐free genome editing (Altpeter *et al*., 2016). In addition, understanding the proteins and processes involved in T‐DNA integration may instruct us about plant DNA repair and recombination processes.

Because incorporating large regions of T‐DNA homology with the plant genome does not result in efficient targeting of T‐DNA molecules for integration into homologous genomic regions (Hanin *et al*., 2001), HR is not a favored mechanism for T‐DNA integration. T‐DNA conversion to double‐strand molecules, followed by ligation into dsDNA breaks, is one popular model for T‐DNA integration (Tinland, 1996; Salomon & Puchta, 1998; Chilton & Que, 2003; Tzfira *et al*., 2003, 2004; Liang & Tzfira, 2013; Dafny‐Yelin *et al*., 2015). Consequently, numerous laboratories have measured stable transformation frequency of rice, tobacco, and *Arabidopsis* NHEJ mutants, or lines in which NHEJ genes have been silenced (Friesner & Britt, 2003; Gallego *et al*., 2003; van Attikum *et al*., 2003; Li *et al*., 2005; Jia *et al*., 2012; Nishizawa‐Yokoi *et al*., 2012; Vaghchhipawala *et al*., 2012; Mestiri *et al*., 2014; Saika *et al*., 2014; Park *et al*., 2015). The results of these studies are conflicting: Some studies indicate that loss of NHEJ gene function results in lower transformation frequencies (Friesner & Britt, 2003; Li *et al*., 2005; Jia *et al*., 2012; Nishizawa‐Yokoi *et al*., 2012; Mestiri *et al*., 2014; Saika *et al*., 2014), others indicate little effect on transformation frequency (Gallego *et al*., 2003; van Attikum *et al*., 2003), and still others found an increase in transformation frequency (Vaghchhipawala *et al*., 2012; Park *et al*., 2015).

As opposed to some of the studies cited above, we have never observed a decrease in stable transformation (relative to transient transformation) when individual NHEJ genes involved in the ‘classical’ (Ku‐dependent) NHEJ pathway are mutated or silenced. Rather, mutation or silencing of some NHEJ genes, including *Ku70*, *Ku80*, *XRCC4*, and *Lig6*, may increase the frequency of stable transformation (Vaghchhipawala *et al*., 2012; Park *et al*., 2015). These studies confirmed that, for these four genes, decreased NHEJ gene expression did not greatly affect the extent of T‐DNA integration when assays were conducted in the absence of selection for transgene expression. Use of these nonselective T‐DNA integration assays is important because T‐DNA integration, a biochemical process that occurs randomly in the host genome (Kim *et al*., 2007), does not always correlate with stable transformation, a phenotype dependent upon transgene expression (Mysore *et al*., 2000b; Crane & Gelvin, 2007; Vaghchhipawala *et al*., 2012; Park *et al*., 2015). Our observations that mutation of up to four plant DNA repair/recombination pathways still results in T‐DNA integration (Fig. 2) suggest that these repair/recombination pathways are not absolutely required for T‐DNA integration.

The interpretation of our results and those of others regarding the importance of DNA polymerase θ (PolQ) in T‐DNA integration is complicated by the observation that PolQ appears to be important for transient as well as stable transformation (Figs 3, 6, 7; van Kregten *et al*., 2016; Kralemann *et al*., 2022; Nishizawa‐Yokoi *et al*., 2021; Nishizawa‐Yokoi & Gelvin, 2023). The level of integrated T‐DNA in the genome of *Arabidopsis polQ* mutants is similar to or approximately half that of WT plants under nonselective experimental conditions, whereas stable transformation, using selective conditions, is considerably more reduced (Figs 3, 4, 6, 7; Nishizawa‐Yokoi *et al*., 2021). Because transient transformation requires conversion of the ss T‐strand to a double‐strand transcription‐competent form but does not require T‐DNA integration, PolQ may be involved in conversion of T‐strands to double‐strand DNA molecules in addition to its role in T‐DNA integration. The increase in both transient and stable transformation observed using *polλ* mutants (Fig. 7) suggests that DNA polymerase λ may inhibit both T‐DNA integration and conversion of T‐strands to a double‐strand form.

Although some experiments of our current study used materials supplied to us by the White laboratory, our results slightly differ from those of Mestiri *et al*. (2014). We note several possible explanations for these disparate results: (1) *Arabidopsis* lines harboring mutations in multiple DNA repair/recombination genes grew poorly, especially under normal light conditions (Fig. S1A). This growth defect may result from accumulated mutations that are not efficiently repaired in these mutant lines, and clearly affects the frequency of transient transformation, a process that does not require T‐DNA integration (Mysore *et al*., 1998). Additional mutations may affect transformation but not growth under low light conditions (Figs S1B, S2). (2) The discrepancy in transient transformation levels may have been caused by different concentrations of *Agrobacterium* used for inoculation. Mestiri *et al*. used a very high *Agrobacterium* inoculum (≥ 10^8^ CFU ml^−1^), a saturating amount of bacteria that would not allow differences in transient transformation frequency to be easily observed. Using a slightly lower bacterial inoculum (10^7^ CFU ml^−1^), we obtained results similar to that of Mestiri *et al*. However, when we further lowered the bacterial inoculum to 10^6^ CFU ml^−1^, differences in transient transformation frequency were readily apparent (Fig. S2A).

The results of van Kregten *et al*. (2016) and Nishizawa‐Yokoi *et al*. (2021) indicate the importance, but not absolute requirement, for DNA polymerase θ in T‐DNA integration and indicate that dsDNA breaks play a role in the transformation process (Levy, 2016). DNA break repair proteins are likely involved in resecting broken DNA ends to provide microhomology with an invading T‐strand, or in ligating double‐strand T‐DNA molecules to plant DNA. Processes that slow DNA break repair may afford more opportunity for T‐strand invasion and copying into resected DNA ends by DNA polymerase θ. Our current results validate our previous conclusions (Vaghchhipawala *et al*., 2012; Park *et al*., 2015) and indicate that simultaneous mutation of several genes involved in multiple different DNA repair and recombination pathways does not completely eliminate stable *Agrobacterium*‐mediated transformation or T‐DNA integration.

Collectively, our results indicate multiple pathways for T‐DNA integration and the likelihood of other plant proteins involved in stable transformation.

## Competing interests

None declared.

## Author contributions

SBG conceived experiments. L‐YL, YS, YK, HS, AN‐Y, DW, WL and SBG conducted experiments. L‐YL and SBG interpreted experiments. SBG wrote the manuscript with help from L‐YL, AN‐Y and HS.

## Disclaimer

The New Phytologist Foundation remains neutral with regard to jurisdictional claims in maps and in any institutional affiliations.

## Supporting information


**Dataset S1** Supporting data sets for Figs 1, 2, 3, 4, 5, 6, 7, and for Figs S1 and S2.

**Fig. S1** Growth of *Arabidopsis* DNA repair/recombination mutants under normal and low light conditions.
**Fig. S2** Transient and stable transformation of DNA repair/recombination mutants grown under low light conditions.
Please note: Wiley is not responsible for the content or functionality of any Supporting Information supplied by the authors. Any queries (other than missing material) should be directed to the *New Phytologist* Central Office.

## Data Availability

Data are presented as Supporting Information for Figs 1, 2, 3, 4, 5c, 6, 7, Figs S1A and S2A.
