# Supplementary material for: Arabidopsis DNA repair mutants can integrate Agrobacterium T‐DNA into the plant genome
Source: New Phytol. 2026 Jun 1;251(4):1811–22. doi: 10.1111/nph.71308 (PMC13373861; doi:10.1111/nph.71308)
Supplement: Supplementary file 1 — Dataset S1 Supporting data sets for Figs 1, 2, 3, 4, 5, 6, 7, and for Figs S1 and S2. [file NPH-251-1811-s002.pdf]

New Phytologist Data Sets

Article title: *Arabidopsis* DNA repair mutants can integrate *Agrobacterium* T-DNA into the plant genome

Authors: Lan-Ying Lee, Yunjia Shen, Yooyoung Kim, Ayako Nishizawa-Yokoi, Hiroaki Saika, Demi White, Wenying Liao, and Stanton B. Gelvin

Article acceptance date: 14 May, 2026

Dataset S1 Supporting datasets for Figures 1-7, and for Figures S1 and S2.

Figure 1
Transient transformation of NHEJ mutants in high light

10^7 cfu/ml

Genotype	# Blue roots	Total roots	% blue roots	Standard error (%)
Col-0 #1	292	300	97.3	
Col-0 #2	182	185	98.4	
Col-0 overall	474	485	98.6	±0.8
ku80 #1	47	56	83.9	
ku80 #2	129	135	95.6	
ku80 #3	74	76	97.4	
ku80 overall	250	267	92.3	±4.2
ku80, xrccl #1	71	83	85.5	
ku80, xrccl #2	53	94	56.4	
ku80, xrccl #3	4856	85.7		
ku80, xrccl overall	172	233	75.9	±9.7
ku80, xrccl, xpf #1	39	41	95.1	
ku80, xrccl, xpf #2	52	55	94.5	
ku80, xrccl, xpf #3	32	36	88.9	
ku80, xrccl, xpf overall	123	132	92.9	±2.0
ku80, xrccl, xpf, xrcc2 #1	23	75	30.7	
ku80, xrccl, xpf, xrcc2 #2	46	55	83.6	
ku80, xrccl, xpf, xrcc2 #3	19	38	50.0	
ku80, xrccl, xpf, xrcc2 overall	88	168	54.8	±15.5

10^6 cfu/ml

Genotype	# Blue roots	Total roots	% blue roots	Standard error (%)
Col-0 #1	155	255	60.8	
Col-0 #2	73	190	38.4	
Col-0 #3	85	140	60.7	
Col-0 overall	313	585	53.3	±7.4
ku80 #1	33	56	58.9	
ku80 #2	46	99	46.5	
ku80 #3	44	70	62.9	
ku80 overall	123	225	56.1	±4.9
ku80, xrccl #1	30	52	57.7	
ku80, xrccl #2	29	73	39.7	
ku80, xrccl #3	48	80	60.0	
ku80, xrccl overall	107	205	52.5	±6.4
ku80, xrccl, xpf #1	17	48	35.4	
ku80, xrccl, xpf #2	13	52	25.0	
ku80, xrccl, xpf #3	14.47	29.8		
ku80, xrccl, xpf overall	44	147	30.1	±3.0
ku80, xrccl, xpf, xrcc2 #1	8	40	20.0	
ku80, xrccl, xpf, xrcc2 #2	2	34	5.9	
ku80, xrccl, xpf, xrcc2 #3	11	53	20.8	
ku80, xrccl, xpf, xrcc2 overall	21	127	15.5	±4.8

Stable transformation of NHEJ mutants in high light

10^7 cfu/ml

Genotype	# Tumors	Total roots	% tumors	Standard error (%)
Col-0 #1	24	81	29.6	
Col-0 #2	26	80	32.5	
Col-0 #3	32	80	40.0	
Col-0-overall	241	82	34.0	±3.1
ku80 #1	21	80	26.3	
ku80 #2	26	77	33.8	
ku80 #3	13	80	16.3	
ku80 overall	237	60	25.4	±5.1
ku80, xrccl #1	23	81	28.4	
ku80, xrccl #2	26	80	32.5	
ku80, xrccl #3	32	81	39.5	
ku80, xrccl overall	242	81	33.5	±3.2
ku80, xrccl, xpf #1	20	80	25.0	
ku80, xrccl, xpf #2	24	80	30.0	
ku80, xrccl, xpf #3	11	80	13.8	
ku80, xrccl, xpf	240	55	22.9	±4.9
ku80, xrccl, xpf, xrcc2 #1	20	81	24.7	
ku80, xrccl, xpf, xrcc2 #2	17	81	21.0	
ku80, xrccl, xpf, xrcc2 #3	9	82	11.0	
ku80, xrccl, xpf, xrcc2	244	46	18.9	±4.1

Figure 2

Droplet digital PCR of NHEJ mutants

	hpt/Act2	hpt/genome	error+	error-	lipA/Act2	lipA/genome	error+	error-
No infection	0.00019	0.000095	0.000225	0.000095	0	0	0	0
Col-0(100)#1	0.022	0.011	0.005	0.005	0.003	0.0015	0.00125	0.00125
Col-0(100)#2	0.0235	0.01175	0.0023	0.0023	0.0046	0.0023	0.0095	0.0095
Col-0(100)#3	0.062	0.031	0.005	0.0045	0.055	0.0275	0.009	0.009
1X(100)#1	0.0064	0.0032	0.0011	0.0011	0.0026	0.0013	0.0055	0.0055
1X(100)#2	0.021	0.0105	0.003	0.003	0.054	0.027	0.0011	0.0011
1X(100)#3	0.123	0.0615	0.0045	0.005	0.043	0.002	0.00175	0.00175
2X(100)#1	0.0073	0.00365	0.00125	0.0013	0.002	0.001	0.0005	0.0005
2X(100)#2	0.0117	0.00585	0.00175	0.00175	0.0047	0.00235	0.00775	0.00775
3X(100)#1	0.0107	0.00535	0.00175	0.0017	0.0033	0.00165	0.000675	0.000675
3X(100)#2	0.0049	0.00245	0.00085	0.0009	0.0022	0.001125	0.000425	0.000425
4X(100)#1	0.0082	0.0041	0.00125	0.0013	0.0038	0.0019	0.000575	0.000575
4X(100)#2	0.0057	0.00285	0.00105	0.00285	0.00295	0.001475	0.00065	0.00065

Figure 3

Transformation of *polQ* and related mutants

Transient transformation
10^8 cfu/ml

Genotype	# Blue roots	Total roots	% blue roots
Col-0 #1	254	286	88.8
Col-0 #2	126	142	88.7
Col-0 combined	380	428	88.8
ku80 #1	280	289	96.9
ku80 #2	249	274	90.1
ku80 combined	529	563	94.0
xrccl #1	303	305	99.3
xrccl #2	311	356	87.4
xrccl combined	614	661	93.0
ku80/xrccl #1	248	341	72.7
ku80/xrccl #2	178	242	73.6
ku80/xrccl combined	426	583	73.1
teb2 #1	125	220	56.8
teb2 #2	164	298	55.0
teb2 combined	289	518	55.8
teb2/ku80 #1	62	148	41.9
teb2/ku80 #2	96	180	53.3
teb2/ku80 combined	157	328	47.9
teb2/ku80/xrccl #1	77	174	44.2
teb2/ku80/xrccl #2	51	127	40.2
teb2/ku80/xrccl combined	128	301	42.5

Stable transformation

10^8 cfu/ml

Genotype	# tumors	Total roots	% tumors
Col-0 #1	98	196	50.0
Col-0 #2	73	185	39.4
Col-0 combined	171	381	44.9
ku80 #1	148	286	51.7
ku80 #2	154	291	52.9
ku80 combined	302	577	52.3
xrccl #1	81	166	48.8
xrccl #2	70	186	37.6
xrccl combined	151	352	42.9
ku80/xrccl #1	77	223	34.5
ku80/xrccl #2	59	221	26.7
ku80/xrccl combined	136	444	30.6
teb2 #1	3	160	1.9
teb2 #2	9	178	5.0
teb2 combined	12	338	3.6
teb2/ku80 #1	6	169	3.6
teb2/ku80 #2	1	128	0.8
teb2/ku80 combined	7	297	2.4
teb2/ku80/xrccl #1	1	170	0.6
teb2/ku80/xrccl #2	1	162	0.6
teb2/ku80/xrccl combined	2	332	0.6

Figure 4 ddPCR of *hptII* and *lipA* in *Arabidopsis* mutants

Well	Sample	Target	Concentrat	CopiesPer2	PoissonCor	PoissonCor	Positives	Negatives
A01	Col1-a	hpt	297	5940	308	292	3101	10795
B01	Col1-b	hpt	377	7540	390	371	3615	9566
C01	Col1-c	hpt	126	2520	133	123	1309	11573
D01	Col1-d	hpt	145	2900	153	141	1328	10108
E01	Col2-a	hpt	80.9	1618	86.5	78.1	828	11625
F01	Col2-b	hpt	206	4120	215	201	1947	10178
G01	Col2-c	hpt	183	3660	191	179	1906	11314
H01	Col2-d	hpt	99.5	1990	105.9	96.3	962	10895
A02	ku80 1-a	hpt	157	3140	164	153	1724	12092
B02	ku80 1-b	hpt	602	12040	618	594	5806	8687
C02	ku80 1-c	hpt	161	3220	168	157	1961	13402
D02	ku80 2-a	hpt	261	5220	271	257	2931	11782
E02	ku80 2-b	hpt	327	6540	338	321	3455	10795
F02	ku80 2-c	hpt	333	6660	344	328	3829	11696
G02	xrccl 1-a	hpt	262	5240	272	257	2743	10975
H02	xrccl 1-b	hpt	663	13260	681	654	5236	6921
A03	xrccl 1-c	hpt	864	17280	883	854	8005	7387
B03	teb2 1-a	hpt	428	8560	441	421	4258	9708
C03	teb2 1-b	hpt	243	4860	251	238	3240	14146
D03	teb2 1-c	hpt	135.4	2708	141.9	132.2	1707	13991
E03	teb2 1-d	hpt	146.5	2930	152.9	143.3	2038	15369
F03	teb2 2-a	hpt	352	7040	363	346	4075	11692
G03	teb2 2-b	hpt	315	6300	325	310	3784	12313
H03	teb2 2-c	hpt	126	2520	133	123	1386	12232
A04	teb2 2-d	hpt	120	2400	127	117	1255	11648
B04	2x 1-a	hpt	202	4040	211	197	2103	11235
C04	2x 1-b	hpt	542	10840	558	534	4669	7973
D04	2x 1-c	hpt	51.7	1034	56	49.4	535	11918
E04	2x 1-d	hpt	133	2660	140	130	1436	11988
F04	2x 2-a	hpt	328	6560	340	322	3186	9898
G04	2x 2-b	hpt	239	4780	248	234	2372	10547
H04	2x 2-c	hpt	134	2680	141	130	1419	11766
A05	2x 2-d	hpt	594	11880	610	585	4944	7535
B05	teb2/ku80 1-a	hpt	225	4500	234	220	2110	10033
C05	teb2/ku80 1-b	hpt	313	6260	325	307	2761	9050
D05	teb2/ku80 1-c	hpt	124	2480	131	120	1326	11944
E05	teb2/ku80 1-d	hpt	91	1820	96.9	88	926	11515
F05	teb2/ku80 2-a	hpt	518	10360	533	510	4625	8361
G05	teb2/ku80 2-b	hpt	107.1	2142	113.1	104	1221	12814
H05	teb2/ku80 2-c	hpt	135	2700	143	132	1381	11320
A06	teb2/ku80 2-d	hpt	68	1360	73.7	65.1	552	9273
B06	teb2/ku80/xrccl	hpt	1239	24780	1267	1225	8669	4642
C06	teb2/ku80/xrccl	hpt	1232	24640	1261	1218	7942	4294
D06	teb2/ku80/xrccl	hpt	223	4460	232	218	2083	9989
E06	teb2/ku80/xrccl	hpt	1226	24520	1256	1211	7065	3851
F06	teb2/ku80/xrccl	hpt	456	9120	470	449	4252	8986
G06	teb2/ku80/xrccl	hpt	294	5880	306	288	2220	7823

Ch1+Ch2+	Ch1+Ch2-	Ch1-Ch2+	Ch1-Ch2-	Linkage	AcceptedD	Ratio	PoissonRat	PoissonRat
3100	1	10794	1	0	13896	0.029	0.034	0.024
3612	3	9553	13	133	13181	0.0477	0.0516	0.0439
1270	39	11173	400	16.6	12882	0.0317	0.0336	0.0298
1293	35	9734	374	40	11436	0.0371	0.0393	0.0348
793	35	10905	720	25.1	12453	0.0245	0.0263	0.0228
1935	12	10046	132	104	12125	0.0395	0.0418	0.0372
1903	3	11240	74	136	13220	0.0303	0.0321	0.0284
947	15	10508	387	54.8	11857	0.025	0.0267	0.0233
1694	30	11585	507	89.2	13816	0.041	0.0432	0.0388
5323	483	7487	1200	204	14493	0.238	0.246	0.23
1930	31	13110	292	42	15363	0.0354	0.0372	0.0335
2889	42	11458	324	118	14713	0.0601	0.0629	0.0574
3399	56	10282	513	205	14250	0.0862	0.0898	0.0826
3756	73	11388	308	82.9	15525	0.0764	0.0796	0.0732
2735	8	10914	61	117	13718	0.0422	0.0446	0.0397
5201	35	6826	95	294	12157	0.124	0.13	0.118
7986	19	7340	47	464	15392	0.135	0.141	0.128
4256	2	9705	3	0	13966	0.046	0.051	0.04
3219	21	14059	87	0	17386	0.0406	0.0427	0.0385
1657	50	13120	871	69.8	15698	0.0406	0.0427	0.0385
1928	110	14468	901	11	17407	0.0438	0.0459	0.0416
3931	144	11204	488	47.6	15767	0.093	0.0966	0.0893
3677	107	11347	966	192	16097	0.099	0.1028	0.0951
1262	124	11083	1149	5.71	13618	0.0453	0.0479	0.0427
1154	101	10753	895	0	12903	0.0399	0.0423	0.0375
2088	15	11117	118	61.1	13338	0.0372	0.0393	0.0351
4669	0	7973	0	0	12642	0.00054	0.00081	0.00027
529	6	11873	45	0	12453	0.008	0.0088	0.0072
1431	5	11950	38	0	13424	0.0197	0.0211	0.0182
3140	46	9731	167	42.1	13084	0.0678	0.071	0.0645
2365	7	10511	36	29.6	12919	0.0356	0.0379	0.0332
1411	8	11670	96	39.8	13185	0.0235	0.0251	0.022
4944	0	7534	1	594	12479	0.053	0.067	0.04
2086	24	9920	113	0	12143	0.0426	0.045	0.0401
2754	7	9016	34	93	11811	0.047	0.0501	0.0439
1276	50	11492	452	0.422	13270	0.0321	0.0341	0.0302
908	18	11309	206	0	12441	0.0193	0.0206	0.0179
4617	8	8324	37	288	12986	0.0777	0.0823	0.0731
1066	155	11047	1767	8.16	14035	0.0458	0.0485	0.043
1338	43	10909	411	18.4	12701	0.0346	0.0366	0.0325
527	25	8810	463	6.16	9825	0.0193	0.021	0.0176
8604	65	4603	39	85.4	13311	0.217	0.227	0.207
7942	0	4294	0	0	12236	0.0012	0.0018	0.0006
2082	1	9982	7	65.7	12072	0.0259	0.0286	0.0231
7057	8	3846	5	102	10916	0.155	0.168	0.142
4232	20	8927	59	112	13238	0.0756	0.0796	0.0717
2213	7	7796	27	22.7	10043	0.0439	0.0471	0.0407

FractionalA	PoissonFra	PoissonFra	Threshold	MeanAmpli	MeanAmpli	MeanAmpli	PoissonCor	PoissonRat
2.78	3.25	2.3	2908	6686.8	1706.9	2818.2	300	0.031
4.56	4.91	4.21	2908	6796.5	1727.7	3117.9	380	0.0497
3.07	3.25	2.89	2908	6960.2	1719.6	2252.1	130	0.0327
3.57	3.78	3.36	2908	7009.5	1720	2334.2	150	0.0382
2.4	2.57	2.23	2908	6972.1	1725.1	2074	84	0.0255
3.8	4.01	3.59	2908	6942.5	1718.6	2557.4	210	0.0406
2.94	3.12	2.76	2908	6764.4	1685.7	2417.9	190	0.0312
2.44	2.6	2.27	2908	7136.4	1750.4	2187.4	100	0.0259
3.94	4.15	3.74	2908	6903.8	1696.2	2346	160	0.0422
19.2	19.7	18.7	2908	7229.6	1704.2	3917.7	610	0.2418
3.42	3.59	3.24	2908	7019.1	1717.4	2394.1	160	0.0363
5.67	5.92	5.43	2908	6988.3	1706.8	2759	270	0.0615
7.94	8.24	7.63	2908	6977	1700.8	2980.1	330	0.0881
7.1	7.37	6.82	2908	7073.1	1710.4	3033	340	0.078
4.04	4.27	3.82	2908	7046.4	1728.5	2791.8	270	0.0434
11.04	11.5	10.58	2908	6946.7	1700.5	3960	670	0.1271
11.9	12.4	11.3	2908	6858.3	1705.4	4385.3	870	0.1381
4.38	4.88	3.89	2908	6845.8	1710	3275.8	430	0.0486
3.9	4.09	3.71	2908	6899.9	1716.7	2682.6	250	0.0416
3.9	4.1	3.7	2908	6886.5	1693	2257.7	140	0.0417
4.19	4.39	4	2908	7171.5	1718	2356.5	150	0.0448
8.5	8.81	8.2	2908	7166.4	1703.8	3115.6	360	0.0948
9	9.32	8.69	2908	7133.6	1722.5	2994.5	320	0.1009
4.33	4.57	4.1	2908	7234	1715	2276.7	130	0.0466
3.84	4.06	3.62	2908	7117	1684	2212.5	120	0.0412
3.59	3.79	3.39	2908	6968.9	1681.3	2515	210	0.0383
0.054	0.081	0.027	2908	5152.7	1729	2993.4	550	0.00081
0.79	0.87	0.71	2908	6342.3	1668.8	1869.6	54	0.00839
1.93	2.07	1.79	2908	6696.7	1693.7	2228.9	140	0.0204
6.35	6.63	6.06	2908	6837.1	1669.8	2928	330	0.0694
3.43	3.65	3.21	2908	6743.8	1675.3	2605.9	240	0.0368
2.3	2.44	2.15	2908	6874.3	1680.4	2239.4	140	0.0243
5.1	6.3	3.9	2908	6680.6	1652.4	3644.5	600	0.06
4.08	4.31	3.86	2908	6855	1656.2	2559.5	230	0.0438
4.49	4.77	4.21	2908	6749.5	1670.9	2858.1	320	0.0486
3.11	3.3	2.93	2908	6821.1	1658.6	2174.4	130	0.0331
1.89	2.02	1.76	2908	6638.6	1630.3	2003.1	94	0.02
7.21	7.61	6.81	2908	6776.8	1650	3475.9	530	0.0801
4.38	4.63	4.13	2908	6954.1	1655.5	2116.5	110	0.0472
3.34	3.53	3.15	2908	6712.5	1627.7	2180.6	140	0.0356
1.89	2.05	1.73	2908	6552.1	1563	1843.3	71	0.0201
17.8	18.5	17.2	2908	7228.6	1690	5297.1	1300	0.222
0.12	0.18	0.06	2908	6966.2	1686.4	5113.4	1200	0.0018
2.52	2.78	2.26	2908	6728.5	1682.7	2553.4	230	0.0273
13.4	14.4	12.4	2908	7026	1683.2	5141.1	1200	0.161
7.03	7.38	6.69	2908	6939.7	1680.6	3369.8	460	0.0777
4.21	4.5	3.91	2908	6587.7	1630.7	2726.4	300	0.0455

PoissonRat	PoissonFra	PoissonFra
0.0261	3.01	2.54
0.0458	4.73	4.38
0.0307	3.17	2.98
0.0359	3.68	3.47
0.0236	2.48	2.31
0.0383	3.91	3.69
0.0293	3.03	2.85
0.0241	2.52	2.36
0.0399	4.05	3.84
0.2337	19.47	18.94
0.0344	3.5	3.33
0.0587	5.8	5.55
0.0844	8.09	7.78
0.0748	7.24	6.96
0.0409	4.16	3.93
0.1212	11.28	10.81
0.1313	12.13	11.6
0.0431	4.63	4.13
0.0395	4	3.8
0.0395	4	3.8
0.0427	4.29	4.09
0.0911	8.66	8.35
0.097	9.16	8.84
0.044	4.45	4.21
0.0387	3.95	3.73
0.0362	3.69	3.49
0.00027	0.081	0.027
0.00759	0.832	0.753
0.019	2	1.86
0.0661	6.49	6.2
0.0344	3.55	3.32
0.0227	2.37	2.22
0.047	5.6	4.5
0.0413	4.2	3.97
0.0454	4.63	4.35
0.0312	3.21	3.02
0.0185	1.96	1.82
0.0754	7.41	7.01
0.0444	4.51	4.25
0.0335	3.44	3.24
0.0184	1.97	1.81
0.212	18.18	17.5
0.0006	0.18	0.06
0.0245	2.65	2.39
0.148	13.9	12.9
0.0736	7.21	6.86
0.0423	4.36	4.06

Well	Sample	TargetType	Target	Concentrat	Supermix	CopiesPer2	PoissonCor	PoissonCor
A07	Col1-a	Ch1Unknov	lipA	79.3	ddPCR Sup	1586	84.2	76.8
B07	Col1-b	Ch1Unknov	lipA	71.6	ddPCR Sup	1432	76.3	69.2
C07	Col1-c	Ch1Unknov	lipA	12.5	ddPCR Sup	250	14.4	11.5
D07	Col1-d	Ch1Unknov	lipA	13.6	ddPCR Sup	272	15.7	12.6
E07	Col2-a	Ch1Unknov	lipA	14.6	ddPCR Sup	292	16.8	13.5
F07	Col2-b	Ch1Unknov	lipA	28.4	ddPCR Sup	568	31.1	26.9
G07	Col2-c	Ch1Unknov	lipA	26.2	ddPCR Sup	524	28.9	24.8
H07	Col2-d	Ch1Unknov	lipA	21.1	ddPCR Sup	422	23.7	19.8
A08	ku80 1-a	Ch1Unknov	lipA	24.5	ddPCR Sup	490	27.6	23
B08	ku80 1-b	Ch1Unknov	lipA	12	ddPCR Sup	240	14.1	10.9
C08	ku80 1-c	Ch1Unknov	lipA	14.4	ddPCR Sup	288	16.7	13.2
D08	ku80 2-a	Ch1Unknov	lipA	58.5	ddPCR Sup	1170	64	55.7
E08	ku80 2-b	Ch1Unknov	lipA	66.4	ddPCR Sup	1328	71.9	63.7
F08	ku80 2-c	Ch1Unknov	lipA	50.6	ddPCR Sup	1012	55	48.4
G08	xrccl 1-a	Ch1Unknov	lipA	32.2	ddPCR Sup	644	35.6	30.4
H08	xrccl 1-b	Ch1Unknov	lipA	23.5	ddPCR Sup	470	26.6	22
A09	xrccl 1-c	Ch1Unknov	lipA	41	ddPCR Sup	820	44.7	39.1
B09	teb2 1-a	Ch1Unknov	lipA	108.8	ddPCR Sup	2176	115.1	105.7
C09	teb2 1-b	Ch1Unknov	lipA	74.4	ddPCR Sup	1488	79.5	71.8
D09	teb2 1-c	Ch1Unknov	lipA	47.9	ddPCR Sup	958	52	45.9
E09	teb2 1-d	Ch1Unknov	lipA	40.3	ddPCR Sup	806	44.2	38.4
F09	teb2 2-a	Ch1Unknov	lipA	72.7	ddPCR Sup	1454	78	70.1
G09	teb2 2-b	Ch1Unknov	lipA	73.6	ddPCR Sup	1472	78.7	71.1
H09	teb2 2-c	Ch1Unknov	lipA	40.9	ddPCR Sup	818	44.5	39
A10	teb2 2-d	Ch1Unknov	lipA	32.2	ddPCR Sup	644	35.3	30.5
B10	2x 1-a	Ch1Unknov	lipA	20	ddPCR Sup	400	22.6	18.6
C10	2x 1-b	Ch1Unknov	lipA	127	ddPCR Sup	2540	134	124
D10	2x 1-c	Ch1Unknov	lipA	16.5	ddPCR Sup	330	19	15.3
E10	2x 1-d	Ch1Unknov	lipA	29	ddPCR Sup	580	32.1	27.4
F10	2x 2-a	Ch1Unknov	lipA	82	ddPCR Sup	1640	87.4	79.2
G10	2x 2-b	Ch1Unknov	lipA	54.3	ddPCR Sup	1086	58.6	52.1
H10	2x 2-c	Ch1Unknov	lipA	28.6	ddPCR Sup	572	31.8	27
A11	2x 2-d	Ch1Unknov	lipA	130	ddPCR Sup	2600	136	126
B11	teb2/ku80	Ch1Unknov	lipA	64.6	ddPCR Sup	1292	69.3	62.1
C11	teb2/ku80	Ch1Unknov	lipA	77.1	ddPCR Sup	1542	82.3	74.5
D11	teb2/ku80	Ch1Unknov	lipA	43.1	ddPCR Sup	862	46.9	41.1
E11	teb2/ku80	Ch1Unknov	lipA	26.9	ddPCR Sup	538	29.9	25.4
F11	teb2/ku80	Ch1Unknov	lipA	101.1	ddPCR Sup	2022	107.2	98.1
G11	teb2/ku80	Ch1Unknov	lipA	25.4	ddPCR Sup	508	28.3	24
H11	teb2/ku80	Ch1Unknov	lipA	47.9	ddPCR Sup	958	51.9	45.9
A12	teb2/ku80	Ch1Unknov	lipA	19.8	ddPCR Sup	396	22.2	18.5
B12	teb2/ku80/	Ch1Unknov	lipA	275	ddPCR Sup	5500	284	270
C12	teb2/ku80/	Ch1Unknov	lipA	281	ddPCR Sup	5620	291	275
D12	teb2/ku80/	Ch1Unknov	lipA	71.7	ddPCR Sup	1434	76.7	69.1
E12	teb2/ku80/	Ch1Unknov	lipA	248	ddPCR Sup	4960	258	243
F12	teb2/ku80/	Ch1Unknov	lipA	103.4	ddPCR Sup	2068	109.4	100.3
G12	teb2/ku80/	Ch1Unknov	lipA	100.4	ddPCR Sup	2008	105.9	97.6

Positives	Negatives	Ch1+Ch2+	Ch1+Ch2-	Ch1-Ch2+	Ch1-Ch2-	Linkage	AcceptedD	Ratio
1031	14786	1031	0	14782	4	79.3	15817	0.0081
893	14224	891	2	14181	43	18.1	15117	0.0105
155	14553	150	5	14190	363	0	14708	0.00287
162	13893	160	2	13488	405	7.84	14055	0.0033
179	14296	163	16	13374	922	0	14475	0.0045
399	16352	397	2	16150	202	16.8	16751	0.0055
357	15866	353	4	15689	177	0	16223	0.0049
255	14097	253	2	13797	300	13.3	14352	0.0046
242	11485	239	3	11049	436	16.5	11727	0.0063
121	11851	110	11	10486	1365	2.51	11972	0.0047
142	11552	139	3	11351	201	0	11694	0.003
429	8418	423	6	8317	101	0	8847	0.0113
574	9881	565	9	9711	170	5.74	10455	0.0139
514	11687	508	6	11565	122	0	12201	0.0094
341	12303	337	4	12175	128	0	12644	0.006
226	11185	226	0	11141	44	23.5	11411	0.0036
476	13414	473	3	13339	75	0	13890	0.0067
1181	12185	1181	0	12172	13	109	13366	0.0133
826	12650	814	12	12364	286	26.1	13476	0.0166
544	13079	531	13	12770	309	0	13623	0.0109
419	12016	390	29	10982	1034	7.78	12435	0.0139
734	11508	685	49	10683	825	4.86	12242	0.0234
820	12696	793	27	11966	730	30.9	13516	0.0217
485	13724	464	21	12664	1060	17.8	14209	0.0135
394	14218	358	36	12500	1718	7.76	14612	0.0129
212	12391	208	4	12186	205	0	12603	0.0041
1262	11058	1262	0	11058	0	0	12320	0.00013
170	12022	170	0	11981	41	16.5	12192	0.00247
329	13198	329	0	13193	5	29	13527	0.00312
881	12204	869	12	11888	316	38.2	13085	0.0189
607	12860	605	2	12812	48	6.23	13467	0.0082
317	12875	317	0	12848	27	28.6	13192	0.00393
1435	12319	1435	0	12318	1	130	13754	0.0116
717	12712	710	7	12597	115	0	13429	0.0117
853	12594	852	1	12539	55	55.9	13447	0.012
487	13060	466	21	12585	475	0	13547	0.0111
312	13490	308	4	13201	289	10.7	13802	0.0059
1083	12064	1080	3	12037	27	0	13147	0.0141
298	13630	259	39	11541	2089	3.68	13928	0.0115
574	13799	568	6	13634	165	5.93	14373	0.0092
253	14914	240	13	14197	717	0	15167	0.0055
3071	11683	3041	30	11478	205	114	14754	0.0564
2870	10653	2868	2	10653	0	0	13523	0.027
775	12335	773	2	12323	12	0	13110	0.0089
2576	10972	2573	3	10937	35	151	13548	0.0359
1122	12217	1115	7	12037	180	58.5	13339	0.0206
1284	14407	1281	3	14391	16	0	15691	0.0127

PoissonRat	PoissonRat	FractionalA	PoissonFra	PoissonFra	Threshold	MeanAmpli	MeanAmpli	MeanAmpli
0.0093	0.007	0.81	0.92	0.7	2886	8471	1064.7	1547.4
0.0113	0.0096	1.04	1.12	0.95	2886	8416	1059.8	1494.4
0.00333	0.00241	0.286	0.332	0.241	2886	8294.1	1074.8	1150.9
0.0038	0.0028	0.33	0.38	0.28	2886	8326.4	1056.9	1140.7
0.0052	0.0039	0.45	0.52	0.39	2886	8325.3	1054	1143.9
0.006	0.0049	0.54	0.6	0.49	2886	8413.1	1064.9	1239.9
0.0055	0.0044	0.49	0.55	0.44	2886	8422	1078.9	1240.5
0.0052	0.0041	0.46	0.52	0.4	2886	8433.8	1072.6	1203.4
0.0072	0.0055	0.63	0.71	0.55	2886	8085.3	1023	1168.7
0.0055	0.0039	0.47	0.55	0.38	2886	8269	1045.6	1118.6
0.0035	0.0025	0.3	0.35	0.25	2886	8286	1043.9	1131.8
0.0124	0.0101	1.11	1.23	1	2886	7878.2	997.05	1330.7
0.0151	0.0126	1.37	1.49	1.25	2886	8063.9	1020.1	1406.8
0.0103	0.0086	0.94	1.02	0.85	2886	8095.1	1019.3	1317.4
0.0067	0.0053	0.6	0.66	0.53	2886	8125.3	1027.7	1219.2
0.0041	0.0031	0.36	0.41	0.31	2886	7818	1038	1172.2
0.0074	0.0061	0.67	0.73	0.6	2886	7834.7	1023.6	1257
0.0146	0.012	1.32	1.44	1.19	2886	8204.3	1018.5	1653.4
0.0178	0.0154	1.63	1.75	1.51	2886	8096.8	1025.1	1458.5
0.0118	0.0099	1.08	1.17	0.98	2886	7835.1	1032.3	1304
0.0153	0.0126	1.37	1.51	1.24	2886	8184.7	1022.8	1264.1
0.0252	0.0216	2.29	2.46	2.12	2886	8246.7	1027.2	1460
0.0233	0.0201	2.13	2.28	1.97	2886	8278.9	1032.6	1472.2
0.0147	0.0122	1.33	1.45	1.21	2886	8161.5	1026	1269.5
0.0142	0.0116	1.27	1.4	1.15	2886	8188.8	1022.6	1215.8
0.0047	0.0036	0.41	0.47	0.36	2886	8141.2	1025.9	1145.6
0.00019	6.00E-05	0.013	0.019	0.006	2886	7100.2	1093.3	1708.6
0.00286	0.00207	0.246	0.285	0.207	2886	7492.3	1049.2	1139.1
0.00361	0.00262	0.311	0.36	0.262	2886	8230.3	1022.2	1197.6
0.0203	0.0175	1.86	1.99	1.72	2886	8284.8	1032.2	1520.5
0.009	0.0075	0.82	0.89	0.74	2886	8211.8	1023.7	1347.7
0.00442	0.00343	0.391	0.44	0.342	2886	8273.3	1024.5	1198.7
0.0145	0.0086	1.14	1.43	0.86	2886	8280.7	1027	1783.8
0.0126	0.0107	1.15	1.25	1.06	2886	8254.1	1030.2	1415.9
0.0129	0.011	1.18	1.28	1.09	2886	8329.3	1050.3	1512.1
0.0121	0.01	1.09	1.2	0.99	2886	8157.3	1032.6	1288.7
0.0066	0.0053	0.59	0.66	0.52	2886	8341.8	1039	1204.1
0.0153	0.0129	1.39	1.51	1.28	2886	8435.2	1037.6	1647
0.0128	0.0102	1.14	1.27	1.01	2886	8331.4	1045.6	1201.5
0.01	0.0084	0.91	0.99	0.83	2886	8526.3	1064.5	1362.5
0.0062	0.0048	0.55	0.62	0.48	2886	8072.3	999.55	1117.5
0.059	0.0537	5.34	5.57	5.1	2886	8391.2	1027.7	2560.4
0.0318	0.0223	2.63	3.09	2.18	2886	8472.3	1036.4	2614.5
0.0098	0.008	0.88	0.97	0.79	2886	8180	1036.1	1458.4
0.0383	0.0335	3.46	3.69	3.24	2886	8606.5	1040	2478.7
0.022	0.0192	2.02	2.15	1.88	2886	8444.1	1035.3	1658.5
0.0138	0.0116	1.26	1.36	1.15	2886	8365.3	1033.9	1633.9

PoissonCor	PoissonRat	PoissonRat	PoissonFra(	PoissonFra(
82	0.0087	0.0076	0.86	0.75
74	0.01091	0.01003	1.079	0.993
13	0.00311	0.00264	0.31	0.263
15	0.00353	0.00301	0.352	0.3
16	0.00489	0.0042	0.487	0.419
30	0.00576	0.00518	0.572	0.516
28	0.00522	0.00468	0.52	0.465
22	0.00494	0.00434	0.492	0.433
26	0.00677	0.00593	0.672	0.589
13	0.00513	0.00427	0.51	0.425
16	0.00328	0.00276	0.327	0.275
61	0.0119	0.0107	1.17	1.05
69	0.0145	0.0133	1.43	1.31
53	0.0099	0.00899	0.98	0.891
34	0.00634	0.00565	0.63	0.562
25	0.00386	0.00334	0.384	0.333
43	0.00707	0.00639	0.702	0.635
110	0.014	0.0127	1.38	1.25
77	0.0172	0.016	1.69	1.57
50	0.01138	0.01039	1.125	1.028
42	0.0146	0.0132	1.44	1.31
75	0.0243	0.0225	2.38	2.2
76	0.0225	0.0209	2.2	2.05
43	0.0141	0.0129	1.39	1.27
34	0.0136	0.0122	1.34	1.21
21	0.00443	0.00385	0.441	0.383
130	0.00019	6.00E-05	0.019	0.006
18	0.00267	0.00226	0.266	0.226
31	0.00336	0.00287	0.335	0.286
85	0.0196	0.0182	1.92	1.79
56	0.00864	0.00785	0.856	0.779
30	0.00418	0.00368	0.416	0.366
130	0.0129	0.0102	1.28	1.01
67	0.01216	0.01118	1.202	1.106
80	0.0125	0.0115	1.231	1.133
45	0.0116	0.0105	1.15	1.04
28	0.00628	0.00559	0.624	0.556
100	0.0147	0.0135	1.45	1.33
27	0.0122	0.0108	1.2	1.07
50	0.00961	0.00878	0.952	0.871
21	0.0059	0.00519	0.587	0.516
280	0.0577	0.055	5.46	5.22
290	0.0294	0.0247	2.86	2.41
74	0.00938	0.00843	0.929	0.836
250	0.0371	0.0347	3.58	3.35
110	0.0213	0.0199	2.09	1.95
100	0.0133	0.0122	1.31	1.2

Figure 5C
Growth of calli on root segments

Genotype	% of root segments with calli	Standard error	p value
**2 weeks callus growth**			
Col-0 #1	81.6		
Col-0 #2	84.2		
Col-0 #3	87.4		
Col-0 combined	84.4	±2.9	
teb2 original #1	73.6		
teb2 original #2	76.0		
teb2 original #3	73.9		
teb2 original combined	74.5	±1.3	0.0059
teb2 crossed #1	76.5		
teb2 crossed #2	72.9		
teb2 crossed #3	80.5		
teb2 crossed combined	76.6	±3.8	0.049
**3 weeks callus growth**			
Col-0 #1	90.2		
Col-0 #2	89.0		
Col-0 #3	95.3		
Col-0 combined	91.5	±3.4	
teb2 original #1	82.5		
teb2 original #2	92.3		
teb2 original #3	87.5		
teb2 original combined	87.4	±4.9	ns
teb2 crossed #1	90.5		
teb2 crossed #2	89.0		
teb2 crossed #3	84.6		
teb2 crossed combined	88.0	±3.1	ns

Mutants are compared to Col-0; ns, not significant

Figure 6
Transformation of *teb2* mutants

Transient transformation
10^7 cfu/ml

Genotype	% blue root segments	Standard error	p value
Col-0 #1	99.7		
Col-0 #2	99.1		
Col-0 #3	99.2		
Col-0 combined	99.3	±0.3	
teb2 (2020) #1	71.2		
teb2 (2020) #2	51.8		
teb2 (2020) #3	63.4		
teb2 (2020) combined	62.1	±9.8	0.0027
teb2 (2024) #1	52.3		
teb2 (2024) #2	64.4		
teb2 (2024) #3	60.4		
teb2 (2024) combined	59.0	±6.0	0.0003
teb2 (crossed) #1	73.5		
teb2 (crossed) #2	61.7		
teb2 (crossed) #3	50.3		
teb2 (crossed) combined	61.8	±11.6	0.005

10^6 cfu/ml

Genotype	% blue root segments	Standard error	p value
Col-0 #1	91.4		
Col-0 #2	88.5		
Col-0 #3	80.6		
Col-0 combined	86.8	±5.6	
teb2 (2020) #1	25.6		
teb2 (2020) #2	10.7		
teb2 (2020) #3	18.6		
teb2 (2020) combined	18.3	±7.5	0.0002
teb2 (2024) #1	12.9		
teb2 (2024) #2	16.8		
teb2 (2024) #3	9.9		
teb2 (2024) combined	13.2	±3.5	0.00004
teb2 (crossed) #1	17.4		
teb2 (crossed) #2	16.9		
teb2 (crossed) #3	11.5		
teb2 (crossed) combined	15.2	±3.3	0.00004

Mutants are compared to Col-0

Stable transformation

10^8 cfu/ml

Genotype	% roots with tumors	Standard error	p value
Col-0 #1	86.1		
Col-0 #2	77.5		
Col-0 #3	76.2		
Col-0 combined	79.9	±5.4	
teb2 (2024) #1	17.9		
teb2 (2024) #2	5.2		
teb2 (2024) #3	21.2		
teb2 (2024) combined	14.8	±8.4	0.0004
teb2 (crossed) #1	13.5		
teb2 (crossed) #2	8.3		
teb2 (crossed) #3	4.9		
teb2 (crossed) combined	8.9	±4.3	0.00006

10^7 cfu/ml

Genotype	% roots with tumors	Standard error	p value
Col-0 #1	82.2		
Col-0 #2	75.8		
Col-0 #3	76.1		
Col-0 combined	78.0	±3.6	
teb2 (2024) #1	0.8		
teb2 (2024) #2	3.0		
teb2 (2024) #3	2.4		
teb2 (2024) combined	2.1	±1.1	0.000004
teb2 (crossed) #1	6.9		
teb2 (crossed) #2	1.6		
teb2 (crossed) #3	0.9		
teb2 (crossed) combined	3.1	±3.3	0.00001

Mutants are compared to Col-0

Figure 7
Transformation of *polQ/polλ* mutants

Transient transformation
10^7 cfu/ml

Genotype	# blue roots	# total roots	% blue roots	Standard error	Tukey's HSD test
Col-0 #1	219	248	88.3		
Col-0 #2	228	273	83.5		
Col-0 #3	120	154	77.9		
Col-0 combined	567	675	84.0	±5.2	a
teb2 #1	135	224	60.3		
teb2 #2	80	129	62.0		
teb2 #3	97	138	70.3		
teb2 combined	312	491	63.5	±5.4	b
polλ #1	128	149	85.9		
polλ #2	124	135	91.9		
polλ #3	137	147	93.2		
polλ combined	389	431	90.3	±3.9	a
teb2/polλ #1	109	166	65.7		
teb2/polλ #2	120	212	56.6		
teb2/polλ #3	110	170	64.7		
teb2/polλ combined	339	548	61.9	±5.0	b

10^6 cfu/ml

Genotype	# blue roots	# total roots	% blue roots	Standard error	Tukey's HSD test
Col-0 #1	77	206	37.4		
Col-0 #2	86	239	36.0		
Col-0 #3	80	240	33.3		
Col-0 combined	243	685	35.5	±2.1	b
teb2 #1	31	165	18.8		
teb2 #2	53	246	21.5		
teb2 #3	37	195	19.0		
teb2 combined	121	606	20.0	±1.5	c
polλ #1	122	153	79.7		
polλ #2	101	177	57.1		
polλ #3	121	200	60.5		
polλ combined	344	530	64.9	±12.2	a
teb2/polλ #1	36	192	18.8		
teb2/polλ #2	60	231	26.0		
teb2/polλ #3	32	174	18.4		
teb2/polλ combined	128	597	21.4	±4.3	c

Mutants are compared to Col-0

Stable transformation

10^8 cfu/ml

Genotype	# roots with tumors	# total roots	% roots with tumors	Standard error	Tukey's HSD test
Col-0 #1	59	116	50.9		
Col-0 #2	58	104	55.8		
Col-0 #3	63	109	57.8		
Col-0 combined	180	329	54.7	±3.6	a
teb2 #1	22	133	16.5		
teb2 #2	16	135	11.9		
teb2 #3	6	109	5.5		
teb2 combined	44	377	11.7	±5.5	b
polλ #1	81	138	58.7		
polλ #2	78	137	56.9		
polλ #3	64	121	52.9		
polλ combined	276	396	69.7	±3.0	a
teb2/polλ #1	3	123	2.4		
teb2/polλ #2	2	106	1.9		
teb2/polλ #3	1	102	1.0		
teb2/polλ combined	6	331	1.8	±0.7	c

10^7 cfu/ml

Genotype	# roots with tumors	# total roots	% roots with tumors	Standard error	Tukey's HSD test
Col-0 #1	56	124	45.2		
Col-0 #2	57	133	42.9		
Col-0 #3	75	146	51.4		
Col-0 combined	188	403	46.7	±4.4	b
teb2 #1	1	104	1.0		
teb2 #2	1	112	1.8		
teb2 #3	2	98	2.4		
teb2 combined	4	314	1.3	±0.6	c
polλ #1	83	149	55.7		
polλ #2	82	153	53.6		
polλ #3	89	149	59.7		
polλ combined	254	451	56.3	±3.1	a
teb2/polλ #1	3	127	2.4		
teb2/polλ #2	2	119	1.7		
teb2/polλ #3	3	101	3.0		
teb2/polλ combined	8	347	2.3	±0.6	c

10^6 cfu/ml

Genotype	# roots with tumors	# total roots	% roots with tumors	Standard error	Tukey's HSD test
Col-0 #1	38	142			
Col-0 #2	44	150			
Col-0 #3	24	151			
Col-0 combined	106	443	23.9	±7.1	b
teb2 #1	2	188			
teb2 #2	0	89			
teb2 #3	1	99			
teb2 combined	3	376	0.8	±0.9	c
polλ #1	67	156			
polλ #2	53	144			
polλ #3	45	129			
polλ combined	165	429	38.5	±4.2	a
teb2/polλ #1	0	109			
teb2/polλ #2	1	110			
teb2/polλ #3	0	112			
teb2/polλ combined	1	331	0.3	±0.5	c

Mutants compared to Col-0

Supplemental Figure S1A
Germination of Seeds of Wild-type and NHEJ mutant plants

	**High Light**	
**Genotype**	**Germination Rate**	**Percentage**
Col-0 (Gelvin)	48/48	100
Col-0 (White)	88/88	100
ku80	63/69	91
ku80, xrccl	47/67	70
ku80, xrccl, xpf	24/44	55
ku80, xrccl, xpf, xrcc2	21/61	34
**Genotype**	**Germination Rate**	**Percentage**
	**Low Light**	
Col-0 (White)	35/35	100
ku80	78/85	92
ku80, xrccl	41/54	76
ku80, xrccl, xpf	67/135	50
ku80, xrccl, xpf, xrcc2	48/173	28

Supplemental Figure S2

Transient transformation of NHEJ mutants in low light

10^7 cfu/ml

Genotype	# Blue roots	Total roots	% blue roots	Standard error (%)
Col-0 #1	3	6	50.0	
Col-0 #2	8	13	61.5	
Col-0 combined	11	19	55.8	±5.7
ku80 #1	32	91	35.2	
ku80 #2	31	54	57.4	
ku80	63	145	46.2	±11.1
ku80, xrccl, xpf #1	12	69	17.4	
ku80, xrccl, xpf #2	6	86	7.0	
ku80, xrccl, xpf combined	18	155	12.2	±5.2
ku80, xrccl, xpf, xrcc2 #1	4	53	7.5	
ku80, xrccl, xpf, xrcc2 #2	2	35	5.7	
ku80, xrccl, xpf, xrcc2 combined	6	88	6.6	±0.91

Stable transformation of NHEJ mutants in low light

10^7 cfu/ml

Genotype	# Tumors	Total roots	% tumors	Standard error (%)
Col-0 #1	36	46	78.3	
Col-0 #2	37	45	82.2	
Col-0 combined	73	91	80.2	±1.4
ku80 #1	41	53	77.4	
ku80 #2	17	20	85.0	
ku80 combined	58	73	81.2	±2.7
ku80, xrccl, xpf #1	20	53	37.7	
ku80, xrccl, xpf #2	13	26	50.0	
ku80, xrccl, xpf #3	17	26	65.4	
ku80, xrccl, xpf combined	50	105	51.0	±8.0
ku80, xrccl, xpf, xrcc2 #1	14	54	25.9	
ku80, xrccl, xpf, xrcc2 #2	19	37	51.4	
ku80, xrccl, xpf, xrcc2 combined	33	91	38.6	±9.0
