# Supplementary material for: Arabidopsis DNA repair mutants can integrate Agrobacterium T‐DNA into the plant genome
Source: New Phytol. 2026 Jun 1;251(4):1811–22. doi: 10.1111/nph.71308 (PMC13373861; doi:10.1111/nph.71308)
Supplement: Supplementary file 2 — Fig. S1 Growth of Arabidopsis DNA repair/recombination mutants under normal and low light conditions. Fig. S2 Transient and stable transformation of DNA repair/recombination mutants grown under low light conditions. Please note: Wiley is not responsible for the content or functionality of any Supporting Information supplied by the authors. Any queries (other than missing material) should be directed to the New Phytologist Central Office. [file NPH-251-1811-s001.pdf]

New Phytologist Supporting Information

Article title: *Arabidopsis* DNA repair mutants can integrate *Agrobacterium* T-DNA into the plant genome

Authors: Lan-Ying Lee, Yunjia Shen, Yooyoung Kim, Ayako Nishizawa-Yokoi, Hiroaki Saika, Demi White, Wenying Liao, and Stanton B. Gelvin

Article acceptance date: 14 May, 2026

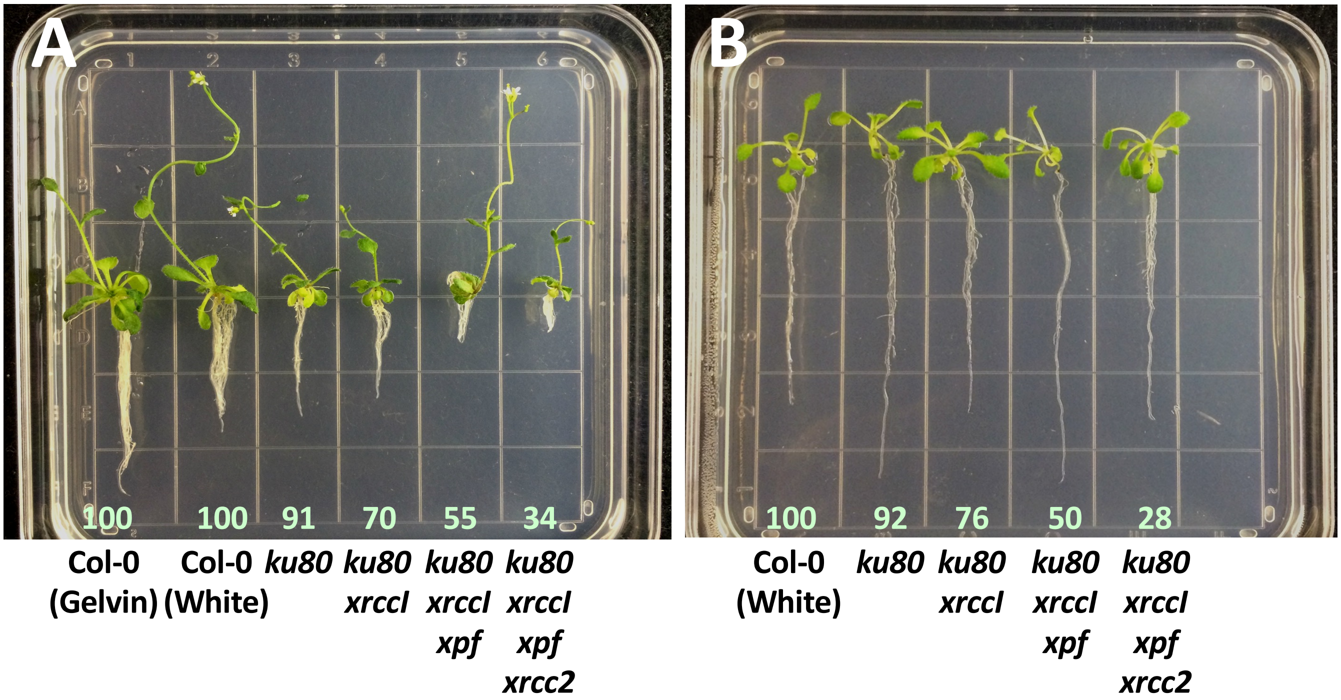

Figure S1. Growth of *Arabidopsis* DNA repair/recombination mutants under high and low light conditions. Seeds were germinated on Gomborg's B5 medium containing 100 µg/ml Timentin, grown vertically under **(A)** high light (~150 µmol/m^2/sec; 16 hr light, 8 hr dark) for 30 days, or **(B)** low light (~9 µmol/m^2/sec; 16 hr light, 8 hr dark) for 15 days, and photographed. The genotypes of each line are indicated below the plate; Col-0 (Gelvin) and Col-0 (White) indicate wild-type Col-0 seeds from the Gelvin and White stock collections. Numbers indicate the % seed germination for each line.

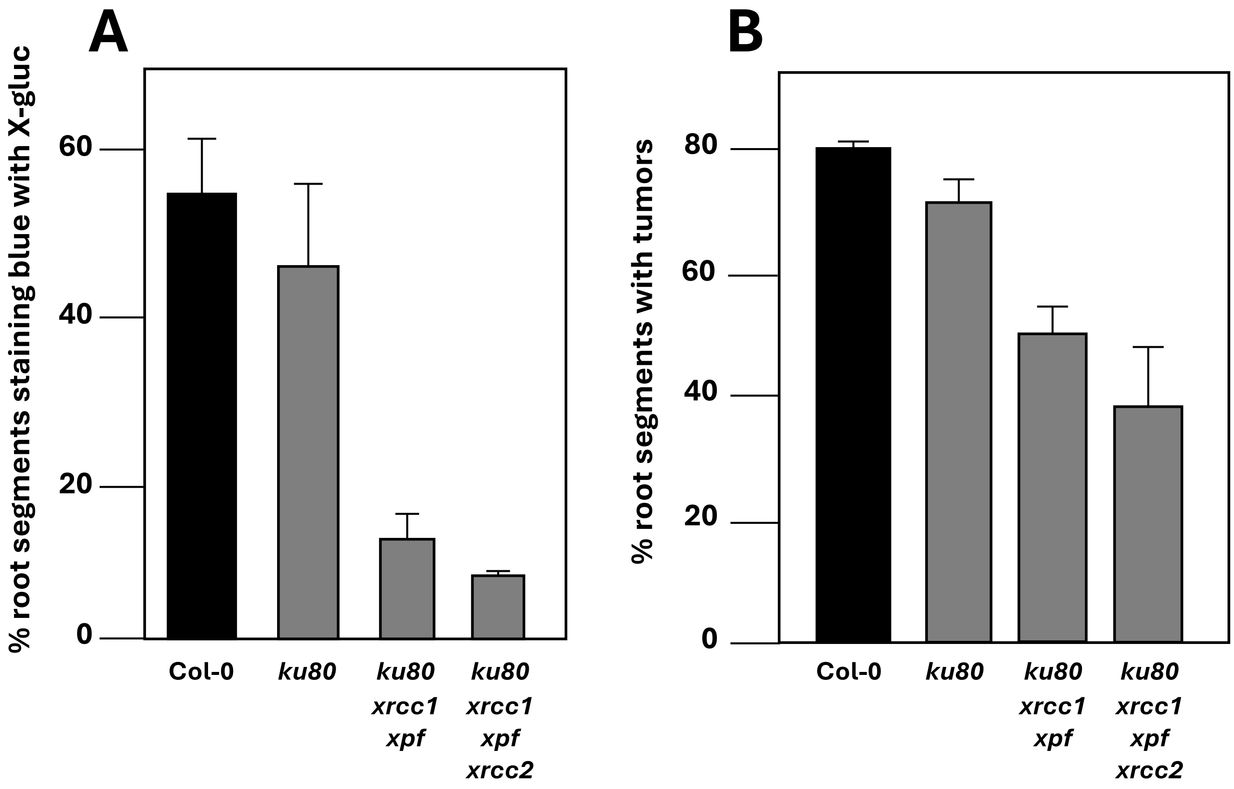

Figure S2. Transient (A) and stable (B) transformation of DNA repair/recombination mutants grown under low light conditions. (A) Segments from pooled roots of 5-10 plants were inoculated with *A. tumefaciens* At849 at a concentration of 10^7 cfu/ml, and incubated on MS medium. After two days, the segments were moved to plates containing CIM plus 100 µg/ml timentin and incubated for an additional four days. Following X-gluc staining, the root segments were visualized using a dissecting microscope and scored for the presence of GUS activity. For each assay point, 90-115 root segments were scored. Wild-type Col-0 seeds were from the White laboratory. **(B)** Segments from pooled roots of 5-10 plants were inoculated with the tumorigenic strain *A. tumefaciens* A208 at a concentration of 10^7 cfu/ml, and incubated on MS medium. After two days, the segments were moved to plates containing MS medium plus 100 µg/ml timentin. Root segments were scored for tumor formation after one month. For each assay point, 120-180 root segments were scored. Wild-type Col-0 seeds were from the White laboratory. For all assays, error bars indicate standard error of the means for two or three replicates.
